# Protein kinase D drives the secretion of invasion mediators in triple-negative breast cancer cell lines

**DOI:** 10.1016/j.isci.2024.108958

**Published:** 2024-01-17

**Authors:** Alexia Gali, Irene V. Bijnsdorp, Sander R. Piersma, Thang V. Pham, Elena Gutiérrez-Galindo, Fiona Kühnel, Nikos Tsolakos, Connie R. Jimenez, Angelika Hausser, Leonidas G. Alexopoulos

**Affiliations:** 1 Biomedical Systems Laboratory, National Technical University of Athens, 15780 Athens, Greece; 2 Protavio Ltd, Demokritos Science Park, 15341 Athens, Greece; 3 Department of Urology, Cancer Center Amsterdam, Cancer Center Amsterdam, Amsterdam UMC, de Boelelaan 1117, Amsterdam 1081 HV, the Netherlands; 4 Department of Medical Oncology, Cancer Center Amsterdam, Amsterdam UMC, OncoProteomics Laboratory, de Boelelaan 1117, , Amsterdam 1081 HV, the Netherlands; 5 Institute of Cell Biology and Immunology, University of Stuttgart, 70569 Stuttgart, Germany; 6 Stuttgart Research Center for Systems Biology, University of Stuttgart, 70569 Stuttgart, Germany

**Keywords:** Cell biology, Cancer

## Abstract

The protein kinase D (PKD) family members regulate the fission of cargo vesicles at the Golgi complex and play a pro-oncogenic role in triple-negative breast cancer (TNBC). Whether PKD facilitates the secretion of tumor-promoting factors in TNBC, however, is still unknown. Using the pharmacological inhibition of PKD activity and siRNA-mediated depletion of PKD2 and PKD3, we identified the PKD-dependent secretome of the TNBC cell lines MDA-MB-231 and MDA-MB-468. Mass spectrometry-based proteomics and antibody-based assays revealed a significant downregulation of extracellular matrix related proteins and pro-invasive factors such as LIF, MMP-1, MMP-13, IL-11, M-CSF and GM-CSF in PKD-perturbed cells. Notably, secretion of these proteins in MDA-MB-231 cells was predominantly controlled by PKD2 and enhanced spheroid invasion. Consistently, PKD-dependent secretion of pro-invasive factors was more pronounced in metastatic TNBC cell lines. Our study thus uncovers a novel role of PKD2 in releasing a pro-invasive secretome.

## Highlights

• PKD2/3 signaling is important for the composition of the TNBC cell secretome
• PKD2 regulates the secretion of six invasion-promoting proteins in TNBC cells
• The PKD2-regulated secretome in TNBC has invasion-promoting properties *in vitro*
• PKD2/3 promote the secretion of TNBC invasion mediators mainly in metastatic cell lines

## Graphical abstract

**Figure:**
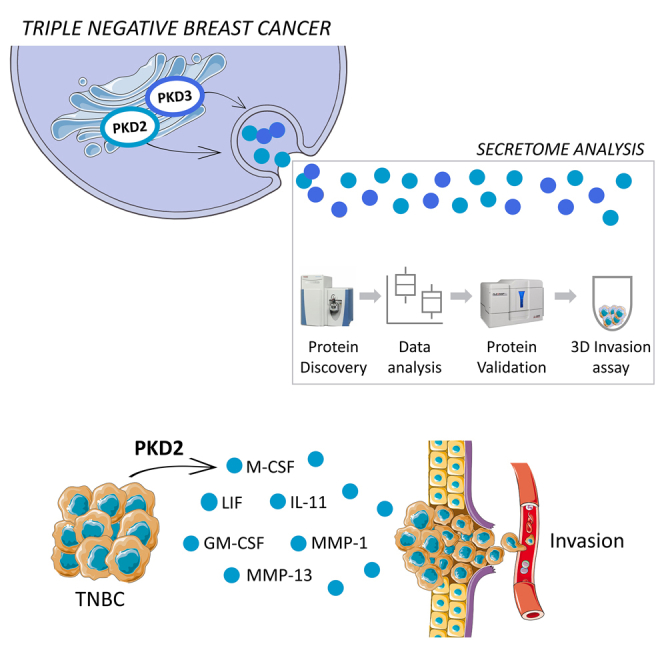

## Introduction

Triple-negative breast cancer (TNBC) is a subtype of breast cancer,^1^^,^^2^ characterized by the lack of expression of estrogen receptors (ER), progesterone receptors (PR) and human epidermal growth factor receptor-2 protein (HER2).^3^ TNBC accounts for 10–20% of all breast cancer cases,^4^ with the primary cause of death being metastatic disease.^5^ Patients with TNBC have poorer overall survival in all stages of the disease, compared to patients diagnosed with other breast cancer subtypes.^6^^,^^7^ Clinical trials for targeted therapies are still ongoing in TNBC, resulting in the availability of few agents and the reliance of patients on cytotoxic chemotherapy.^8^

One of the mechanisms partly responsible for the metastatic potential of TNBC is the deregulation of the secretory pathway.^9^ Alterations in the secretome during TNBC progression enable autocrine and paracrine signaling between different cell subtypes in the tumor microenvironment (TME),^10^ cell-extracellular matrix (ECM) interactions, ECM remodeling,^11^ degradation of the basement membrane and formation of a pre-metastatic niche in distant organs,^12^ among others. Several signaling molecules of the secretory pathway are hijacked during cancer development,^13^ among them protein kinase D (PKD).^14^

The PKD family of serine-threonine protein kinases consists of three isoforms, PKD1, PKD2 and PKD3, which are best known for controlling secretion by participating in vesicle fission at the trans-Golgi network (TGN)^15^^,^^16^ and additionally participate in actin remodeling during cell migration.^17^ In TNBC, the main isoforms present are PKD2 and PKD3, following the epigenetic silencing of PKD1 during breast tumor progression.^18^ To date, several studies have linked PKD3 levels to increased proliferation, cell motility, invasion and cancer stem cell maintenance^19^^,^^20^^,^^21^^,^^22^^,^^23^^,^^24^ and PKD2 to drug resistance, cell adhesion and migration.^25^^,^^26^ Both PKD2 and PKD3 isoforms have also been described as positive regulators of epithelial to mesenchymal transition (EMT) in TNBC.^22^ Notably, the PKD2-and PKD3-regulated secretomes have exhibited pro-invasive and pro-motility properties in prostate and pancreatic cancer.^27^^,^^28^ Hence, it could be hypothesized that PKD contributes to TNBC secretion and may regulate the secretion of pro-oncogenic factors, therefore driving tumor progression.

In the present study, we investigated the PKD2 and PKD3-dependent secretome in TNBC cell lines using quantitative mass spectrometry (MS)-based proteomics (label-free GeLC-MS/MS) and antibody-based multiplex assays. We discovered secreted proteins previously characterized as ECM regulators and invasion mediators in TNBC and validated the PKD-regulated secretion of leukemia inhibitory factor (LIF), matrix metalloproteinase-1 (MMP-1), matrix metalloproteinase-13 (MMP-13), interleukin-11 (IL-11), colony-stimulating factor (M-CSF) and granulocyte-macrophage-colony-stimulating factor (GM-CSF), connecting PKD activity to a pro-tumorigenic secretome. Based on evidence obtained from the MDA-MB-231 cell line, we describe a predominantly PKD2-driven effect in the secretion of invasion mediators, with a smaller contribution from PKD3, and demonstrate that the PKD2-regulated secretome promotes invasion *in vitro*. Our results also show that PKD signaling regulates the secretion of a greater number of invasion mediators in established TNBC cell lines derived from metastatic sites than in TNBC cell lines derived from the primary tumor. Thus, we provide evidence that PKD2 and PKD3 regulate the secretion of specific pro-invasive cargo proteins associated with TNBC invasion.

## Results

### Protein kinase D contributes to the composition of the triple-negative breast cancer secretome

**Figure 1:**
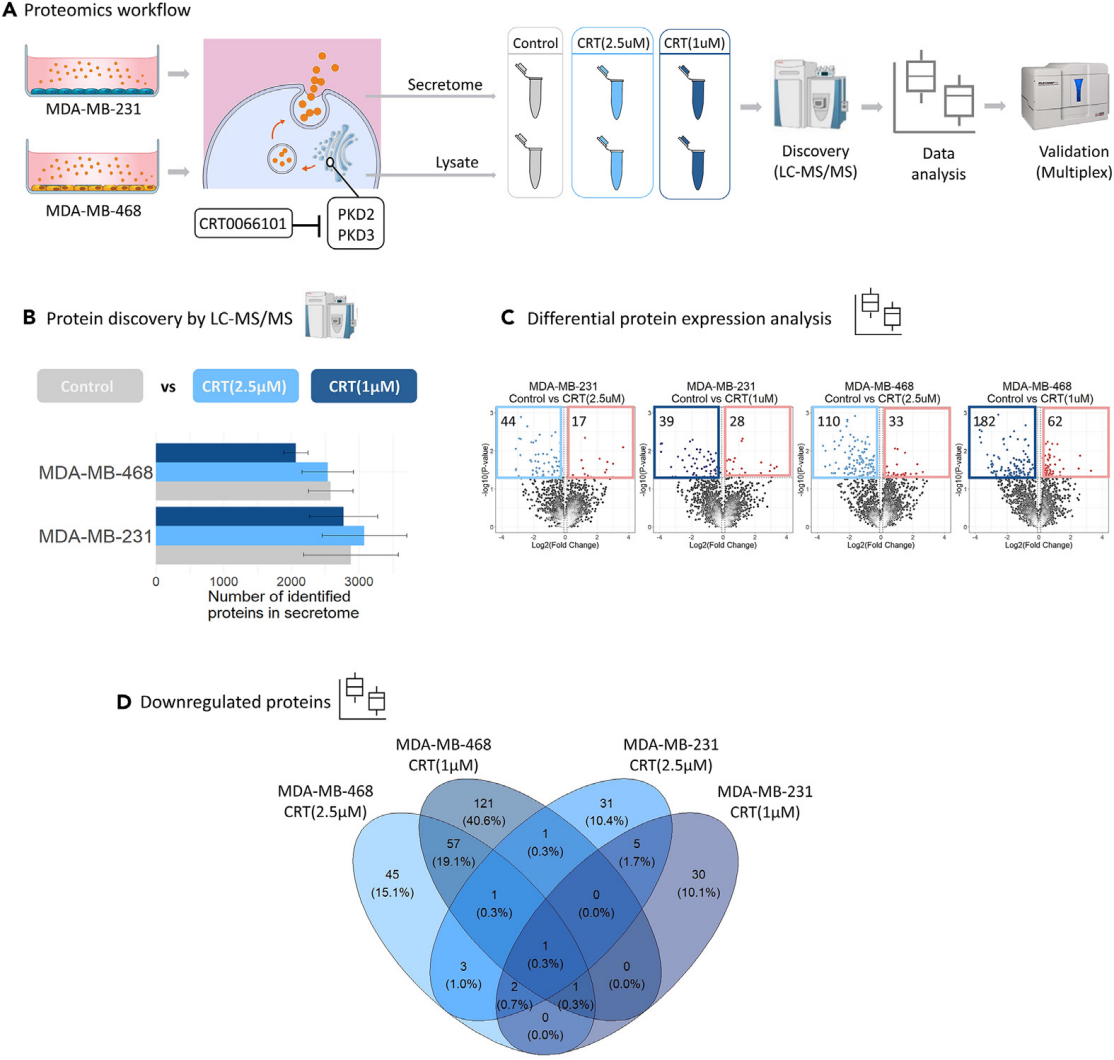
PKD contributes to the composition of the TNBC secretome (A) Overview of the proteomics workflow. Conditioned media (secretome) and cell lysates from PKD-inhibited MDA-MB-231 and MDA-MB-468 cells were collected for LC-MS/MS analysis. Samples were analyzed by 1D-SDS-PAGE, with each gel lane cut into 3 bands, in-gel digested (IGD) with trypsin and analyzed by LC-MS/MS. Identified proteins were quantified by spectral counting and selected proteins with significantly reduced expression were further examined. A panel of selected proteins was validated using antibody-based multiplex assays. (B) Number of proteins identified by LC-MS/MS in the secretome samples collected from each cell line and treatment condition, in three biological replicates. Numbers are reported as the mean of the three biological replicates and error bars show standard deviation. (C) Identification of significantly changed secretome proteins upon CRT(2.5μΜ) and CRT(1μΜ), compared to control, per cell line. Volcano plots of log10 p values (statistical significance) against log2 protein abundance fold change between CRT(2.5μΜ) and CRT(1μΜ)-treated cells versus control for each cell line. In light and dark blue are downregulated secretome proteins upon CRT(2.5μΜ) and CRT(1μΜ), respectively, compared to control (log2FoldChange ≤ − 0.1 and p value ≤0.05). Number of downregulated proteins per inhibition scheme and cell line are indicated. Upregulated proteins following both CRT(2.5μΜ) and CRT(1μΜ) are found in red (log2FoldChange ≥0.1 and p value ≤0.05). (D) Venn diagram showing the overlap of downregulated proteins among the four comparisons. See also Figure S1.

We hypothesized that PKD2 and PKD3 regulate the secretion of proteins in TNBC that have a pro-oncogenic role. To test this, we selected two TNBC cell lines, MDA-MB-231 and MDA-MB-468, which are PKD2/PKD3 positive and PKD1 negative and treated both cell lines with the selective pan-PKD inhibitor CRT0066101 (CRT).^21^ Pharmacological inhibition of PKD2 and PKD3 was performed with 2.5 μΜ CRT0066101 (here referred to as CRT (2.5μΜ)) for 2 h and 1 μΜ CRT0066101 (here referred to as CRT (1μΜ)) for 8 h under serum free conditions, followed by recovery of the cells in serum free medium for a total of 24 h from the start of treatment (Figure 1A). The conditioned medium (referred to here as “secretome”), and respective cell lysates, were analyzed to identify the proteins whose secretion to the extracellular space is regulated by PKD2 and PKD3 in TNBC.

PKD2 and PKD3 activity was reduced by ∼ 60–80% following pharmacological inhibition, as verified by monitoring the autophosphorylation level (pS876) (Figures S1A–S1C). Sampling at 24 h allowed enough time for secretome changes to become evident^29^ and cell viability was not affected under serum free conditions in control and CRT treated cells, as confirmed by a CellTiter-Glo luminescent cell viability assay (Promega) (Figure S1D). For secretome profiling, we employed a label-free GeLC-MS/MS-based proteomics workflow^30^ (Figure 1A), analyzing 3 biological replicates for each sample in the treatment and control groups per cell line.

Proteomic profiling of secretomes by LC-MS/MS identified an average of ∼2,700 proteins in MDA-MB-231 samples and ∼2,000 proteins in MDA-MB-468 samples (Figure 1B; Table S1). In MDA-MB-231 cells, both CRT (2.5μΜ) and CRT (1μΜ) induced a significant change in the levels of approximately 60 proteins, whereas in MDA-MB-468 the same inhibition schemes significantly affected the levels of 143 and 244 proteins, respectively (Table S2). Most of the significantly changed proteins were downregulated in response to CRT (2.5μΜ) and CRT(1μΜ), in both cell lines, with 44 and 39 proteins, respectively, found in MDA-MB-231 cells and 110 and 182 showing reduced secretion in MDA-MB-468 cells (Figure 1C). Eight proteins were significantly downregulated upon both MDA-MB-231 CRT (2.5μΜ) and CRT (1μΜ), whereas 60 were found to overlap between MDA-MB-468 CRT (2.5μΜ) and CRT (1μΜ) (Figure 1D) (Table S3). The levels of overlap in both cell lines were significant at p < 0.001 (one-sided Fisher’s exact test). Notably, protein overlap within the same cell line was more evident after CRT treatment.

### Protein kinase D activity induces the secretion of triple-negative breast cancer invasion mediators

OutCyte analysis^31^ was used to interrogate the class of downregulated secretome proteins identified from both cell lines. Out of the total 299 downregulated proteins, 170 (56.8%) contained a signal peptide, 26 (8.7%) a transmembrane domain and 79 (26.4%) proteins were classified as intracellular. Finally, a total of 24 (8%) proteins were predicted to be unconventionally secreted by the algorithm (Figure S2A). Our analysis confirmed that PKD-dependent protein cargo is, in its majority, classically secreted, which is in agreement with the kinase’s role in regulating constitutive secretion from the TGN.^16^^,^^32^

**Figure 2:**
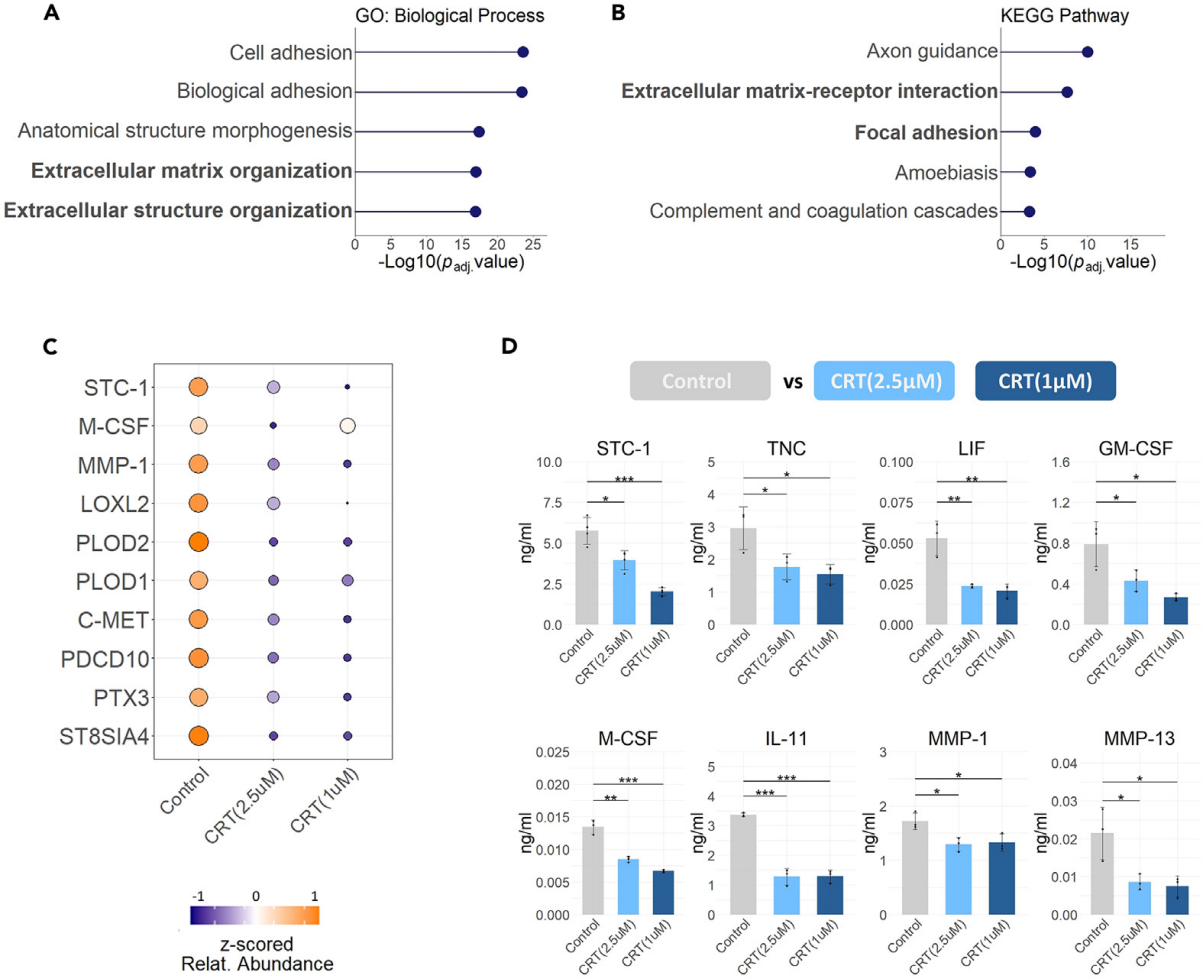
PKD inhibition reduces the secretion of TNBC invasion mediators (A) Top GO biological processes and (B) top KEGG pathways for the downregulated proteins identified in MDA-MB-231 and MDA-MB-468 cells following CRT(2.5μΜ) and CRT(1 μM). X axis indicates the enrichment scores [-log10 (adjusted p value), p value cut-off of 0.05] for each term and y axis the enriched term. (C) Heatmap of Z-scores for relative protein abundances of downregulated invasion mediators found in MDA-MB-231 secretome samples by LC-MS/MS. Each column represents the mean of three biological replicates (n = 3). Relative protein abundances are shown from low (blue) to high (orange). (D) Validation of eight selected secretome proteins in the MDA-MB-231 cells as PKD-regulated. Protein levels (ng/mL) were quantified in secretome samples using multiplex assays for STC-1, LIF, GM-CSF, M-CSF, IL-11, MMP-1, MMP-13 and ELISA for TNC. Data are reported as mean of three or four biological replicates and error bars show standard deviation. p values were assessed by unpaired, two-tailed Student’s *t* test. ∗p < 0.05; ∗∗p < 0.01; ∗∗∗p < 0.001. See also Figures S2–S5.

Following GO enrichment we confirmed that the proteins downregulated in the secretomes of both cell lines were, in nature, extracellular proteins (adjusted p value = 1.67 × 10^−43^), and proteins of the ECM (adjusted p value = 8.76 × 10^−24^) (Figure S2B) with roles in “cell adhesion” (adjusted p value = 2.82 × 10^−24^) and the “extracellular matrix organization” (adjusted p value = 1.23 × 10^−17^) (Figures 2A, S2C, and S2D). Similarly, the two most enriched KEGG pathways to which the downregulated proteins belonged were “Axon guidance” (adjusted p value = 9.18 × 10^−11^) and the “Extracellular matrix-receptor interaction” (adjusted p value = 2.19 × 10^−8^) (Figures 2B, S2E, and S2F).

Downregulated proteins involved in cell adhesion comprised of semaphorins (SEMA3A, SEMA3C, SEMA3E, SEMA3F, SEMA4B, SEMA4D), their primary receptors neuropilins (NRP1) and plexins (PLXNA1, PLXNB2), as well as ephrins (EFNA5, EFNB3) and their Eph receptors (EPHA4, EPHB2, EPHB4, EPHB6) (Figure S3). ECM proteins identified as downregulated in the secretome belonged to various classes including glycoproteins, proteoglycans, ECM regulators and secreted factors, as per Naba et al..^33^^,^^34^ Amongst them were multiple invasion mediators that have been previously described to promote invasion and metastasis in TNBC, specifically, matrix metalloproteinase-1 (MMP-1), macrophage colony stimulating factor (M-CSF), tenascin-C (TNC), leukemia inhibitory factor (LIF), stanniocalcin-1 (STC-1), collagen posttranslational modification proteins lysyl oxidase (LOX), lysyl oxidase homolog 2 (LOXL2) and procollagen-lysine,2-oxoglutarate 5-dioxygenase 2 (PLOD2)^35^^,^^36^^,^^37^^,^^38^^,^^39^^,^^40^^,^^41^^,^^42^^,^^43^^,^^44^^,^^45^^,^^46^^,^^47^^,^^48^^,^^49^^,^^50^^,^^51^^,^^52^^,^^53^^,^^54^^,^^55^^,^^56^^,^^57^^,^^58^ (Figure 2C). By contrast, proteins with increased secretion after PKD inhibition in the two TNBC cell lines did not have an antagonist role in invasion, as examined by enrichment analysis, but were instead associated with various metabolic processes (Figure S4).

The identification of invasion mediator proteins in our dataset suggested a potential mechanism upon which PKD exerts its pro-invasive role in TNBC through its kinase activity. We therefore focused our subsequent work on elucidating the role of the different PKD isoforms on the regulation of the secretion of these mediators. We first validated our proteomic results for STC-1, TNC, LIF, M-CSF and MMP-1 using antibody-based methods (multiplex assays and ELISA). The proteins were only quantified in the secretome samples and not in the cell lysates during mass spectrometry analysis, due to low abundance (Table S4). We included in the validation set three additional proteins, granulocyte-macrophage-colony-stimulating factor (GM-CSF), interleukin 11 (IL-11) and matrix metalloproteinase-13 (MMP-13), which were not identified in the secretome samples during LC-MS/MS, possibly due to their low abundance, but have an established role in TNBC progression.^37^^,^^59^^,^^60^^,^^61^^,^^62^^,^^63^^,^^64^

Validation was initially performed on the MDA-MB-231 cell line which exhibited the most ECM protein enriched dataset (Figure S2C) and under the same conditions used for proteomic profiling. Samples from cells recovering under serum-free and serum-containing (10% fetal bovine serum) conditions were tested to account for the potential effect of serum deprivation on PKD activity, and therefore the regulation of protein secretion. Equal protein content of cell lysates pre and post treatment was confirmed by BCA analysis before secretome sample analysis by multiplex assays and ELISA (Figure S5A). Reduced the secretion of STC-1, TNC, LIF, GM-CSF, M-CSF, IL-11, MMP-1 and MMP-13 in the MDA-MB-231 cell line was confirmed under serum containing conditions (Figure 2D), as well as partially under serum-free conditions (Figure S5B) after PKD inhibition in both treatment schemes, CRT (2.5μΜ) and CRT (1μΜ).

To understand if the reduced secretion of the validated proteins upon PKD inhibition was a result of altered protein expression or differential secretion, we assessed the intracellular levels of these proteins following antibody-based analysis (multiplex assays and ELISA) of the respective cell lysates. The concentration of the secreted proteins was lower intracellularly than in the secretome, as expected.^65^ The intracellular protein levels of STC-1, TNC, M-CSF, MMP-1 and MMP-13 were downregulated upon CRT (2.5μΜ) or CRT(1μΜ) or both inhibition schemes. In contrast, the levels of LIF, GM-CSF and IL-11 were unaltered in the cell lysates following PKD inhibition (Figure S6).

### Protein kinase D2, and to a lesser extent Protein kinase D3, regulates the secretion of triple-negative breast cancer invasion mediators in MDA-MB-231 cells

**Figure 3:**
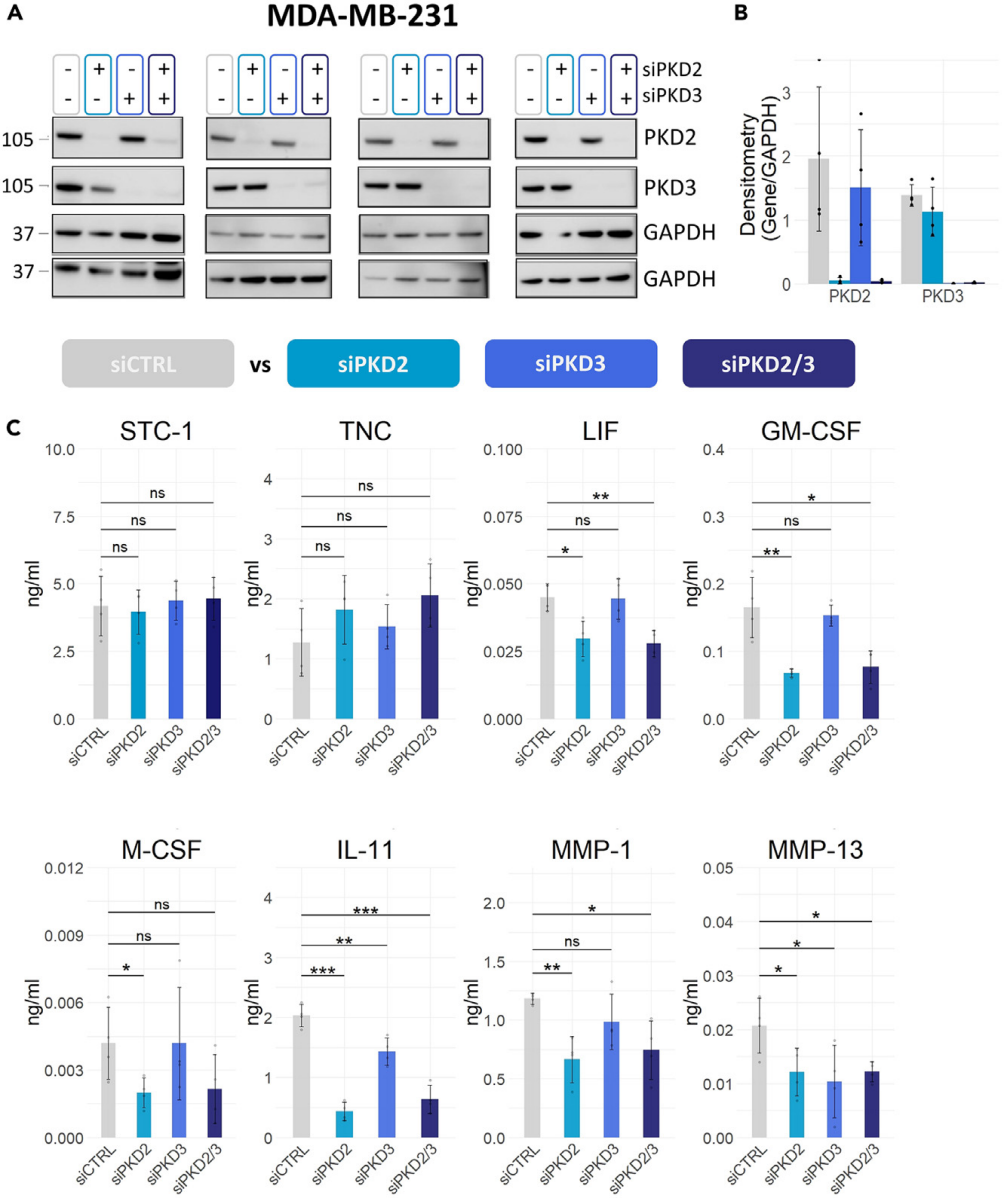
PKD2, and to a lesser extent PKD3, regulates the secretion of TNBC invasion mediators in MDA-MB-231 cells (A and B) Quantification of PKD2 and PKD3 protein expression levels in MDA-MB-231 cells following siRNA-mediated PKD2, PKD3 or PKD2 and PKD3 knockdown (siPKD2, siPKD3, siPKD2/3), compared to cells transfected with a non-targeting control siRNA (siCTRL). Representative immunoblots of four independent experiments are shown. (C) Protein levels (ng/mL) were quantified in secretome samples using multiplex assays for STC-1, LIF, GM-CSF, M-CSF, IL-11, MMP-1, MMP-13 and ELISA for TNC. Data are reported as the mean of four biological replicates and error bars show standard deviation. p values were assessed by unpaired, two-tailed Student’s *t* test. ∗p < 0.05; ∗∗p < 0.01; ∗∗∗p < 0.001; ns: not significant. See also Figure S7.

To investigate the isoform-specific regulation of secretion and confirm that the effect observed is not due to reported off-target effects of the inhibitor,^66^ MDA-MB-231 cells were transiently transfected with non-targeting control siRNA (siCTRL), PKD2 siRNA (siPKD2), PKD3 siRNA (siPKD3), and both PKD2 and PKD3 siRNA (siPKD2/3), and their secretome was analyzed for the previously validated set of proteins STC-1, TNC, LIF, GM-CSF, M-CSF, IL-11, MMP-1 and MMP-13. Knockdown efficiency of PKD2 and PKD3 was confirmed by immunoblotting (Figures 3A and 3B, uncropped blots in Figure S7). Equal protein content of cell lysates amongst knockdown conditions was confirmed by BCA analysis before the respective secretome samples were analyzed by multiplex assays and ELISA (Figure S7A).

With the exception of STC-1 and TNC, PKD2 knockdown and dual PKD2/PKD3 knockdown reduced the secretion of the tested proteins compared to the non-targeting siRNA control (Figure 3C). PKD3 knockdown reduced the secretion of IL-11 by 1.4-fold and of MMP-13 by 2-fold but had no effect on the other proteins. No additive effect was observed upon double PKD2 and PKD3 knockdown on the secretion of IL-11 and MMP-13. These results suggest that PKD2 is the predominant isoform driving the secretion of invasion mediators in TNBC, with only a minor contribution from PKD3. Additionally, the reduced secretion of STC-1 and TNC upon pharmacological inhibition could not be replicated by the knockdown study suggesting it may have been an off-target effect of the inhibitor.

### The Protein kinase D2-regulated secretome promotes the invasion of MDA-MB-231 spheroids

Following our observation that PKD2 is the predominant isoform driving the secretion of invasion mediators in TNBC cell lines, we assessed the invasion-promoting properties of the PKD2-regulated secretome in a 3D cell culture model. MDA-MB-231 cells were transiently transfected with non-targeting control siRNA (siCTRL), and PKD2 siRNA (siPKD2), and their conditioned media was collected and applied to a 3D invasion assay of MDA-MB-231 spheroids embedded in a collagen plug. Knockdown efficiency of PKD2 was confirmed by immunoblotting (Figure S8A, uncropped blots in Figure S8B).

**Figure 4:**
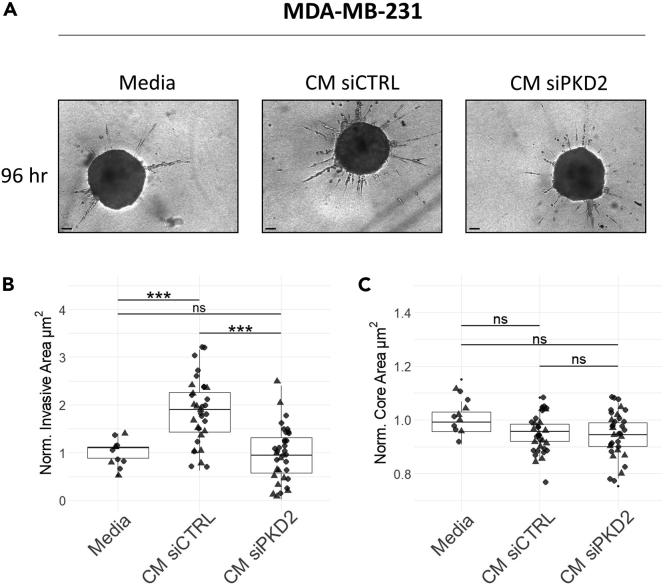
The PKD2-regulated secretome promotes the invasion of MDA-MB-231 spheroids (A) Representative images of MDA-MB-231 spheroids in a 3D invasion assay after 96 h of incubation in serum-containing media, conditioned media (CM) from siCTRL transfected cells and CM from siPKD2 transfected cells. (B and C) Boxplots showing the quantification of B) invasive area and C) core area after 96 h of incubation in growth media (n = 11 spheroids), CM of siCTRL (n = 33 spheroids) and CM of siPKD2 (n = 35 spheroids). The data were normalized by dividing the invasive or core area of each spheroid to the respective average value of medium-treated spheroids (n = 11). Data are reported as dots from two independent experiments. Scale bars, 100 μm. p values were assessed by one way ANOVA (Tukey’s multiple comparisons) ∗p < 0.05; ∗∗p < 0.01; ∗∗∗p < 0.001; ns: not significant. See also Figure S8.

Conditioned media of siCTRL cells strongly enhanced the invasion of spheroids compared to normal growth media suggesting that pro-invasive factors were secreted by MDA-MB-231 cells. By contrast, spheroids treated with conditioned media from siPKD2 cells showed significantly lower invasive capacity comparable to that of spheroids treated with growth media. (Figure 4). Notably, the spheroid core area was comparable across conditions throughout the treatment, indicating that cell proliferation was unaffected. These data complement our previous findings on invasion mediators being present in the PKD2-regulated secretome.

### Protein kinase D regulates the secretion of invasion mediators predominantly in metastatic triple-negative breast cancer cell lines

To gain a systems level understanding of PKD contribution to TNBC secretion, we assessed the effect of PKD inhibition on the previously validated invasion mediators in an expanded panel of TNBC cell lines consisting of 6 cell lines originally established from the primary tumor and 4 metastatic cell lines from pleural effusions^67^^,^^68^^,^^69^^,^^70^^,^^71^ (Table S5). PKD2 and PKD3 were expressed in all cell lines with varying intracellular levels as observed by immunoblotting (Figure S9A, uncropped blots in Figure S10). These results are in line with public expression data acquired from DepMap (Public 22Q4) (Table S5). Treatment with CRT(2.5μΜ) was sufficient to block PKD activity in all cell lines albeit to a different extent as verified by the decrease in the phosphorylation of pS876 in PMA-stimulated cells. (Figures S9A and S9B).

**Figure 5:**
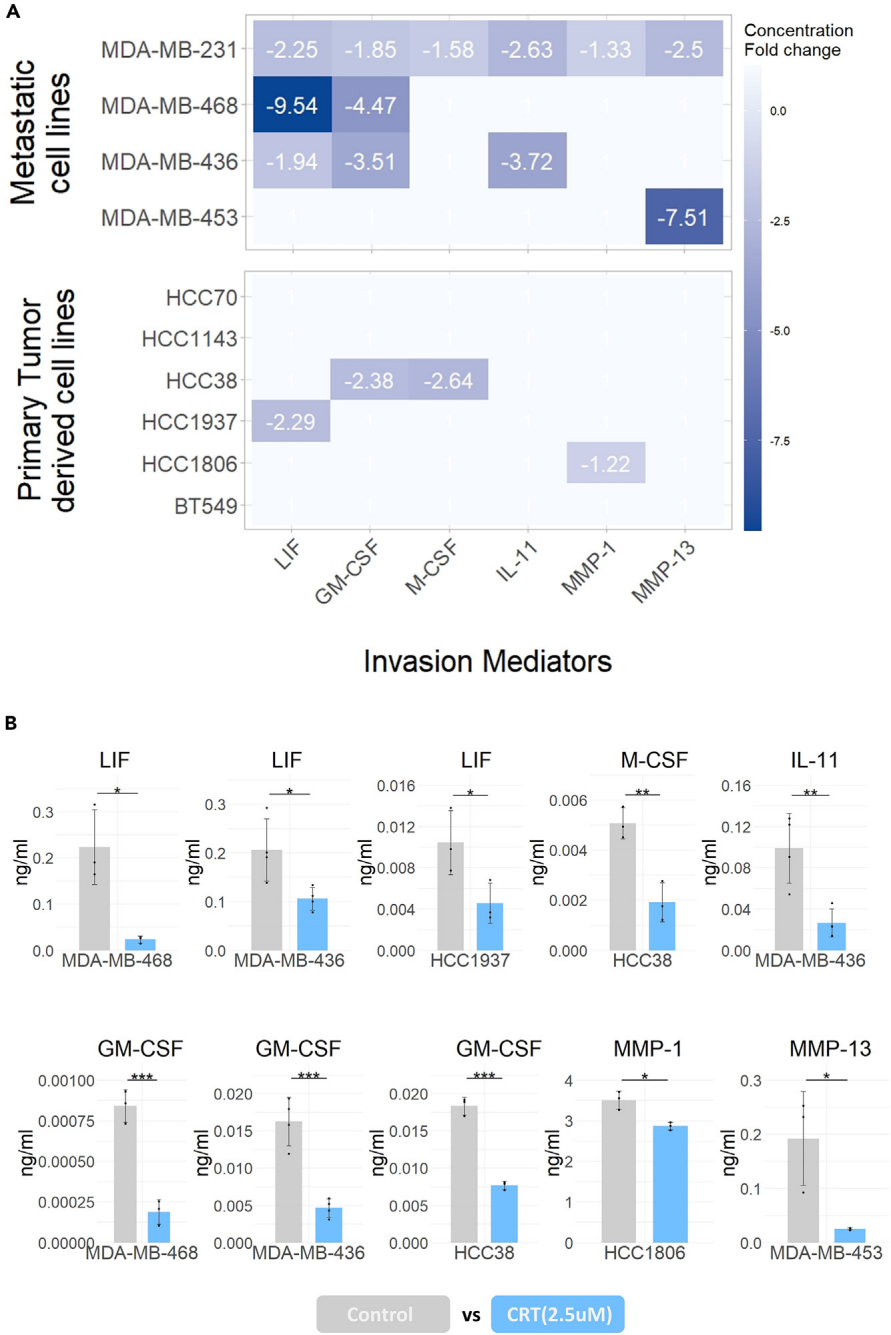
PKD regulates the secretion of invasion mediators predominantly in TNBC metastatic cell lines (A) Heatmap of protein concentration fold change values (CRT(2.5μΜ)/control ≤ − 1.2) for the eight invasion mediators measured per cell line following PKD inhibition by CRT(2.5μΜ). Cell lines are ordered by the site from which they were originally established (metastasis or primary tumor). (B) Protein levels (ng/mL) that showed significant reduction following CRT(2.5μΜ) in the TNBC cell line panel. LIF, GM-CSF, M-CSF, IL-11, MMP-1 and MMP-13 were quantified in secretome samples using multiplex assays. Data acquired from three or four independent biological replicates and error bars show standard deviation. Significance established at p ≤ 0.05 by unpaired, two-tailed Student’s *t* test. ∗p < 0.05; ∗∗p < 0.01; ∗∗∗p < 0.001. See also Figures S9–S11 and Table S5.

Results from the analysis of invasion mediators LIF, M-CSF, GM-CSF, IL-11, MMP-1 and MMP-13 in the secretome samples from the CRT(2.5μΜ)-treated cell lines are presented in Figure 5A. Equal protein content of cell lysates before and after treatment was confirmed by BCA analysis prior to secretome samples being analyzed by multiplex assays and ELISA (Figure S11A). PKD signaling was found to drive the secretion of invasion mediators predominantly in cell lines originally established from pleural effusions and therefore metastasized. This was evident based on both the greater number of mediators affected and magnitude of the effect (fold-change) in these cell lines. Specifically, LIF and GM-CSF secretion was PKD-dependent in the metastatic cell lines MDA-MB-231, MDA-MB-468 and MDA-MB-436, compared to one primary tumor cell line while the secretion of IL-11 and MMP-13 was found to be regulated by PKD only in metastatic TNBC cells (Figures 5A and 5B). PKD had no effect on the secretion of some of these proteins on one or more cell lines despite their quantifiable levels in the secretome of these cells (Figure S11B). Protein measurements in the secretome of TNBC cell lines that showed statistically significant reduction following CRT(2.5μΜ) can be found in Figure 5B.

## Discussion

Protein kinase D is a fundamental kinase of the trans-Golgi network regulating vesicle fission and trafficking. Recent studies have highlighted that PKD2 and PKD3 are involved in signaling pathways linked to tumor progression in different cancer subtypes, including TNBC,^18^^,^^20^^,^^21^^,^^25^^,^^27^^,^^28^^,^^72^^,^^73^ however, the role of PKDs in regulating TNBC progression has not yet been associated with the secretion of pro-oncogenic factors. Here, we have identified PKD kinase signaling to be important for the composition of the TNBC cell secretome and provide a comprehensive understanding of the secreted proteins regulated by the kinases. Proteomic analysis, including LC-MS/MS and antibody-based assays, revealed that LIF, GM-CSF, M-CSF, IL-11, MMP-1 and MMP-13 are invasion-promoting factors whose secretion is regulated in a PKD-dependent manner in TNBC. Secretion of STC-1 and TNC was also reduced upon the pharmacological inhibition of PKD with CRT0066101, however, it was unchanged in PKD2/PKD3 depleted MDA-MB-231 cells. Therefore, we cannot exclude an off-target effect of the kinase inhibitor with respect to the secretion of these two proteins. Nevertheless, the effect of short-term PKD inhibition by CRT0066101 may not be consistent with the phenotype of a three-day PKD knockdown in terms of secretion regulation of these two factors.^74^ Further experiments with CRT0066101 on PKD knockdown cells may provide more insight into whether the secretion of STC-1 and TNC is an off-target effect or whether the cytokines are secreted by compensatory mechanisms after PKD knockdown.

Two different PKD inhibition schemes were implemented to assess the effect on secretome upon acute and prolonged PKD inhibition. This approach indicated that the protein sets affected by the different schemes, although largely different in number and protein identity, are involved in similar processes and pathways as evident by pathway enrichment analysis. The observed differences in protein expression profiles could be attributed to different recovery times but also be linked to inherently diverse cell adaptation mechanisms. Nevertheless, the implementation of the two inhibition schemes allowed us to identify common pathways that we could link to specific protein targets present in either CRT(2.5μΜ), CRT(1μΜ) or both inhibition schemes.

PKD family members regulate the fission of cargo vesicles at the Golgi complex, therefore regulating classical protein secretion^16^^,^^32^; this was reflected in the secretome samples analyzed by LC-MS/MS as the majority of the identified downregulated proteins contained a signal peptide. In an early TNBC study, inhibition by CRT0066101 reduced migration, invasion and metastasis,^21^ an effect later linked with reduced protein levels of MMP-9 and other EMT-related factors in cellular lysates of MDA-MB-231 cells.^22^ Our discovery that the PKD-regulated secretome contains proteins involved in the organization of the ECM and cell adhesion is therefore in line with the previously described PKD functions.

We hypothesized that PKD invasive functions could be exerted by secreted proteins with a previously described role in TNBC invasion, hence selecting for validation a panel of eight such proteins. The factors identified by mass spectrometry, combined with evidence from previous literature, suggest that PKD is a contributing factor in ECM remodeling occurring during TNBC invasion. The collagenases we found to be secreted in a PKD-regulated manner, MMP-1 and MMP-13, are released by tumor cells to degrade both the basement membrane and the ECM to allow cancer cell invasion and metastasis to distant organs.^35^^,^^36^^,^^37^^,^^38^^,^^39^^,^^59^^,^^60^^,^^75^ In pancreatic cancer cell lines, PKD2 is known to regulate the secretion of MMP-2, MMP-7 and MMP-9 via a multiprotein complex with ARF1 and ARL1, and Arfaptin2,^28^^,^^76^ aiding the invasion of cells *in vitro* and *in vivo*.^28^ Similarly, in prostate cancer cell lines, PKD3 contributed to the composition of the secretome by regulating the secretion of MMP-9^27^ and the expression of urokinase-type plasminogen activator (uPA),^73^ both factors involved in ECM remodeling.^77^^,^^78^ Furthermore, conditioned medium from PKD3 knockdown pancreatic cancer cell lines reduced the migration of control cells, indicating the presence of secreted factors in the PKD3-regulated secretome that stimulate cell motility.^27^

The tumor microenvironment supports not only ECM remodeling but also the presence of inflammation in the surrounding tissue.^79^^,^^80^ Tumor-secreted factors have been found to recruit various immune cell types to promote a tumorigenic microenvironment. We have identified the immunomodulatory cytokines M-CSF and GM-CSF, which have been found to act in an autocrine and/or paracrine way to promote TNBC invasion,^42^^,^^43^^,^^64^ as two factors whose secretion is regulated by PKD in different TNBC cell lines. Secretion of GM-CSF has been strongly associated with PKD signaling, with evidence that it is regulated in the MDA-MB-231, MDA-MB-468, MDA-MB-436 and HCC38 cell lines. Recent work in prostate cancer identified that PKD2 or PKD3 knockdown results in decreased expression and secretion of pro-inflammatory chemokines SCF, CCL5 and CCL11 in two prostate cancer cell lines, with the proteins contributing to the chemotactic migration of mast cells *in vitro*.^81^ In another study in prostate cancer, PKD3 knockdown was responsible for the reduced secretion of pro-inflammatory cytokines IL-6, IL-8, and GROα.^27^ Our findings may thus suggest that PKD signaling contributes to immune cell recruitment in the TNBC tumor microenvironment as well.

Not limited to matrix metalloproteinases (MMPs) and pro-inflammatory cytokines, we identified that pharmacological inhibition as well as the depletion of PKD2 regulates the secretion of invasion mediators and IL-6 family members, LIF and IL-11. In the MDA-MB-231 cell line, exogenous LIF treatment was found to promote invasion and metastasis.^48^ Additionally, knockdown of LIF decreased the expression levels of mesenchymal markers Vimentin and N-cadherin, suggesting that LIF can promote EMT.^50^ Secretion of LIF could, therefore, exert PKD2’s pro-invasive^21^^,^^26^ and pro-EMT functions^22^ in an autocrine and/or paracrine manner. As an activator of osteoclast differentiation,^82^ IL-11 has been an important factor for breast cancer metastasis to the bone.^62^^,^^63^^,^^83^^,^^84^^,^^85^ CRT0066101 treatment in an MDA-MB-231 mouse model reduced the number and size of lymph node and lung metastases. The study, however, did not assess bone metastasis.^21^ In prostate cancer, on the other hand, decreased invasion and expression of genes related to bone metastasis was observed upon PKD2/3 knockdown or CRT0066101 inhibition. Additionally, reduced bone metastasis of prostate cancer cells was observed upon CRT0066101 treatment.^86^ The identification of IL-11, as well as of MMP-1 and MMP-13, which are also mediators of breast cancer metastasis to the bone,^37^^,^^87^ supports the hypothesis that PKD2 and PKD3 contribute, via the secretome, to the colonization of the bone in TNBC.

Our data indicate a previously unknown function of PKD2 in TNBC secretion that can be linked to invasion, based on secretome data obtained from MDA-MB-231 cells. PKD2 has so far been associated with drug resistance in breast cancer^25^ and has been found to be located at focal adhesions in MDA-MB-231 cells, contributing to cell adhesion and migration.^26^ We validated the PKD2-regulated secretion of LIF, GM-CSF, M-CSF, IL-11, MMP-13 and MMP-1 and the reduced secretion of IL-11 and MMP-13 upon PKD3 knockdown. These results indicate isoform-specific rather than redundant functions of PKD2 and PKD3 in the secretion of pro-invasive proteins. Complementary to the proteomic data, we observed the invasion-promoting properties of the PKD2-regulated secretome in a 3D invasion assay. Therefore, it can be hypothesized that the proteins we validated to be secreted in a PKD2-dependent manner act in concert to establish this invasive cell behavior. Although it has been suggested that PKD2 and PKD3 may have similar pro-invasive functions in TNBC,^21^ a body of literature has been highlighting PKD3 as the main isoform driving motility and invasion.^19^^,^^20^^,^^21^^,^^23^ Our findings suggest that PKD2 also contributes to TNBC invasion via the kinase-dependent secretome, which is distinct from that of PKD3.

The identification of multiple invasion mediators in the PKD-regulated secretome suggests that the kinases may be contributing to different stages of TNBC invasion, from ECM remodeling to extravasation and finally to metastatic colonization. Importantly, we demonstrated on a systems level that PKD signaling has a greater role in the secretion of invasion mediators in TNBC cell lines established from metastatic sites, providing evidence from a panel of ten cell lines with different sites of origin. Our findings suggest that as cells become more capable of invasion and eventually metastasis, PKD plays a greater role in the secretion of TNBC invasion mediators, presumably to allow tumor progression. Therefore, inhibiting PKD activity by CRT0066101 could limit the secretion of pro-invasive proteins in TNBC cells, with the effect more evident in metastatic cell lines.

Not limited to ECM proteins, our proteomics dataset contained several cell adhesion-annotated proteins that were downregulated following PKD inhibition. Interestingly, SEMA3C was the only protein downregulated in both cell lines with both CRT treatments. Currently, the protein’s role remains contradictory in the literature, with reports finding SEMA3C to have a tumor suppressor role in breast cancer^88^ and others associating it with tumor growth and invasion.^89^ Future research may uncover the functions this secreted axon guidance molecule triggers in a PKD-dependent manner and whether it contributes to the regulation of cell motility and/or invasion by the kinase.

Overall, the results of this study increase our understanding of the contribution of PKD2 and PKD3 to the composition of the TNBC secretome and indicate that the kinases employ the secretory pathway in an isoform-specific manner to support invasive cell behavior.

### Limitations of the study

LC-MS/MS methods are inherently limited in detecting small proteins that are present in low concentrations in secretome samples. This may have led to an underrepresentation of other relevant secreted mediators in the final dataset. This could also partly explain the small overlap of significantly decreased secretome proteins from the CRT(2.5μΜ) and CRT(1μΜ) inhibition schemes (Figure 1D). Further experiments with CRT0066101 on PKD knockdown cells could uncover if reduced the secretion of STC-1 and TNC following PKD inhibition was an off-target inhibitor effect. Transcriptional analysis can also indicate if the PKD-dependent secretion of the validated invasion mediators is a result of altered protein expression or differential secretion, complementing therefore our preliminary findings using intracellular protein data.

## STAR★Methods

### Key resources table

**Table:** 

REAGENT or RESOURCE	SOURCE	IDENTIFIER
**Antibodies**		

Anti-GAPDH	Cell Signaling Technology	Cat# 97166; RRID: AB_2756824
Anti-Tubulin	Merck Millipore	Cat#05–829; RRID:AB_310035
Anti-Phospho-PKD/PKCμ-(Ser916)-PKD	Cell Signaling Technology	Cat#2051; RRID:AB_330841
Anti-Phospho-(Ser744/748)-PKD	Cell Signaling Technology	Cat# 2054; RRID:AB_2172539
Anti-PKD3	Cell Signaling Technology	Cat# 5655; RRID:AB_10695917
Anti-PKD2	Cell Signaling Technology	Cat# 8188; RRID: AB_10829368
anti-Rabbit IgG	Jackson ImmunoResearch Labs	Cat# 111-035-144; RRID:AB_2307391
anti-Mouse IgG	Jackson ImmunoResearch Labs	Cat# 115-035-062; RRID:AB_2338504

**Chemicals, peptides, and recombinant proteins**		

CRT0066101	Tocris	Cat# 4975
Lipofectamine™ RNAiMAX	Thermo Fisher Scientific	Cat# 13778030
PMA (Phorbol 12-myristate 13-acetate)	Merck Millipore	Cat# P1585
PdBu (Phorbol 12,13-dibutyrate)	Tocris	Cat# 4153

**Critical commercial assays**		

Human Tenascin-C ELISA Kit	Abcam	Cat# ab213831
CellTiter-Glo	Promega	Cat# G7570
Pierce™ BCA Protein Assay Kit	Thermo Fisher Scientific	Cat# 23225

**Deposited data**		

Mass Spectrometry Proteomics Data	ProteomeXchange Consortium via PRIDE	PXD041042

**Experimental models: Cell lines**		

MDA-MB-231	CLS	RRID:CVCL_0062
MDA-MB-468	CLS	RRID:CVCL_0419
MDA-MB-453	A kind gift from Prof. Dr. Tilman Brummer (University of Freiburg)	RRID:CVCL_0418
MDA-MB-436	A kind gift from Prof. Dr. Tilman Brummer (University of Freiburg)	RRID:CVCL_0623
HCC70	A kind gift from Prof. Dr. Tilman Brummer (University of Freiburg)	RRID:CVCL_1270
HCC38	A kind gift from Prof. Dr. Tilman Brummer (University of Freiburg)	RRID:CVCL_1267
HCC1143	A kind gift from Prof. Dr. Tilman Brummer (University of Freiburg)	RRID:CVCL_1245
HCC1937	A kind gift from Prof. Dr. Tilman Brummer (University of Freiburg)	RRID:CVCL_0290
HCC1806	ATCC	RRID:CVCL_1258
BT549	CLS	RRID:CVCL_1092

**Oligonucleotides**		

spNon: ON-Target plus non-targeting control pool	Dharmacon	Cat# D-001810–10
spPKD3: ON-Target plus SMARTpool human PRKD3	Dharmacon	Cat# L-005029
spPKD2: ON-Target plus SMARTpool human PRKD2	Dharmacon	Cat# L-004197

**Software and algorithms**		

R Project for Statistical Computing	R Foundation for Statistical Computing	RRID:SCR_001905
ImageJ	ImageJ	RRID:SCR_003070
xPONENT® Software	Luminex Corporation –A DiaSorin Company	https://int.diasorin.com/en/licensed-technologies/reagents-accessories/software
g:Profiler (version e104_e.g.,51_p15_2719230)	ELIXIR Recommended Interoperability Resource^90^	https://biit.cs.ut.ee/gprofiler/gost
Enrichr (library KEGG_2021_Human)	Ma’ayan Laboratory, Icahn School of Medicine at Mount Sinai^91^	https://maayanlab.cloud/Enrichr/
OutCyte	Molecular Proteomics Laboratory, Heinrich-Heine-University Dusseldorf^31^	http://www.outcyte.com/
IncuCyte® S3	Sartorius AG	RRID:SCR_023147

**Other**		

DMEM high glucose	Thermo Fisher Scientific	Cat# 41965-039
RPMI-1640	Thermo Fisher Scientific	Cat# 21875-034
Fetal Bovine Serum (FBS)	Gibco	Cat# 10270106
Penicillin-Streptomycin	Thermo Fisher Scientific	Cat# 15140-122
DMSO	MP Biomedicals	Cat# 11DMSO0001
NuPage Novex 4–12% Bis-Tris gels	Thermo Fisher Scientific	Cat# NP0336
NuPAGE LDS Sample Buffer	Thermo Fisher Scientific	Cat# NP0007
NuPAGE MES SDS Running Buffer	Thermo Fisher Scientific	Cat# NP0002
NuPAGE Antioxidant	Thermo Fisher Scientific	Cat# NP0005
PageRuler™ Prestained Protein Ladder, 10 to 180 kDa	Thermo Fisher Scientific	Cat #26616
iBlot™ Transfer Stack, nitrocellulose	Thermo Fisher Scientific	Cat# IB301001
Blocking Reagent	Roche	Cat# 11096176001
PBS	Gibco	Cat# 10010015
TWEEN 20	Sigma-Aldrich	Cat# P9416
Lysis buffer	Protavio Ltd	N/A
Protease Inhibitors	Protavio Ltd	N/A
PMSF (Phenylmethylsulfonyl fluoride)	MilliporeSigma	Cat#P7626
SuperSignal™ West Pico PLUS Chemiluminescent Substrate	Thermo Fisher Scientific	Cat# 34580
Nunclon™ Sphera™ 96-Well, Nunclon Sphera-Treated, U-Shaped-Bottom Microplate	Thermo Fisher Scientific	Cat# 174929
Corning® Matrigel® Growth Factor Reduced (GFR) Basement Membrane Matrix	Corning	Cat# 356231
FibriCol® Type I Collagen Solution	FibriCol, Cell Systems	Cat# 5133

### Resource availability

#### Lead contact

Further information and requests for resources and reagents should be directed to the lead contact, Leonidas G. Alexopoulos (leo@mail.ntua.gr).

#### Materials availability

This study did not generate new unique reagents.

#### Data and code availability

• The mass spectrometry proteomics data are publicly accessible and have been deposited to the ProteomeXchange Consortium via the PRIDE partner repository with the dataset identifier PXD041042. The rest of the data generated during this study are graphically represented in this article and its supplemental information files.
• This paper does not report original code.
• Any additional information required to reanalyze the data reported in this paper is available from the lead contact upon request.

### Experimental model and study participant details

MDA-MB-231 (RRID:CVCL_0062), MDA-MB-436 (RRID:CVCL_0623), MDA-MB-453 (RRID:CVCL_0418) cells were cultured in DMEM low glucose (Gibco, USA, #41965-039). MDA-MB-468 (RRID:CVCL_0419), BT549 (RRID:CVCL_1092), HCC1806 (RRID:CVCL_1258), HCC38 (RRID:CVCL_1267), HCC70 (RRID:CVCL_1270), HCC1143 (RRID:CVCL_1245) and HCC1937 (RRID:CVCL_0290) cells were cultured in RPMI-1640 (Gibco, USA, #21875-034). All media were supplemented with 10% fetal bovine serum (Gibco, USA, #10270106) and 1% penicillin/streptomycin (Life Technologies, USA, #15140-122). All cell lines were cultured at 37°C in a humidified chamber with 5% CO_2_ and were authenticated in the last three years by SNP profiling.

### Method details

#### Immunoblotting

Western blot was conducted as previously reported.^92^ In short, cells were lysed, samples were loaded on NuPage Novex 4–12% Bis-Tris gels (Thermo Fisher Scientific, USA, #NP0336) and proteins were blotted using the iBlot system (Thermo Fisher Scientific, USA, #IB301001). Sources and dilutions used for the following antibodies were: 1:1,000 for PKD2 (Cell Signaling, USA, #8188, RRID: AB_10829368), 1:2,000 for PKD2 pS876 (https://doi.org/10.1083/jcb.200110047), 1:1,000 for PKD3/PKCν (Cell Signaling, USA, #5655, RRID:AB_10695917), 1:1,000 for PKD3 pS744/748 (Cell Signaling, USA, #2054, RRID:AB_2172539), 1:1,000 for GAPDH (Cell Signaling, USA, #97166, RRID: AB_2756824), 1:1,000 for tubulin (Merck Millipore, USA, #05–829, RRID:AB_310035), 1:10,000 for HRP goat anti-mouse (Jackson ImmunoResearch Labs, USA, #115-035-062, RRID:AB_2338504) and 1:10,000 for HRP goat anti-rabbit (Jackson ImmunoResearch Labs, USA, #111-035-144, RRID:AB_2307391). Membranes were incubated with specific primary antibodies and proteins were visualized with HRP-secondary antibodies. Densitometry was performed using the ImageJ software (ImageJ, RRID:SCR_003070). Uncropped immunoblots are shown in Figures S6 and S8.

#### Cell viability measurements

Cell viability analysis was performed using the CellTiter-Glo luminescent cell viability assay (Promega, USA, #G7570), according to manufacturer’s instructions.

#### Sample preparation of secretomes and cell lysates for mass spectrometry

Conditioned medium and cell lysate from cell lines was collected and processed as previously described.^93^ For mass spectrometry, 1 million MDA-MB-231 or MDA-MB-468 cells were seeded in 60 mm dishes in serum-complete medium without the presence of antibiotics and were allowed to reach ∼90% confluency over 24 h. Cells were subsequently washed three times with PBS and treated with 2 mL of (a) serum free medium for 24 h (b) 2.5 μM CRT0066101 (referred here as CRT(2.5μΜ)) in serum free medium for 2 h and replacement with serum free medium for 22 h (c) 1 μM CRT0066101 (referred here as CRT(1μΜ)) in serum free medium for 8 h and replacement with serum free medium for 16 h. The pan-PKD inhibitor CRT0066101 was purchased from Tocris Bioscience (Biotechne, USA, #4975). The inhibition schemes were selected to assess an acute inhibition, using 2.5 μΜ of the inhibitor for a short period of 2 h, versus a longer inhibition, using a smaller dose at 1 μM over a longer period of 8 h. Following confirmation of PKD activity reduction by immunoblotting, treatment media was replaced with fresh serum-free media to assess the effects of PKD inhibition in the secretome and cell lysate. Conditioned medium (secretome) was collected and centrifuged (15 min at 500 x g at 4°C followed by 20 min at 2000 x g at 4°C) for the removal of cell debris. The supernatant was concentrated to ∼30 μL by passing over an Amicon 3 kDa filter (Merck Millipore, USA, #UFC500324). For the collection of cell lysates, cells were washed three times with ice-cold PBS and scraped following the addition of LDS-sample buffer/b-mercaptethanol. Lysed cells were centrifuged at 14000 x g for 15 min at 4°C and kept at - 80°C until further analysis.

#### Gel electrophoresis and in-gel digestion of proteins

Gel electrophoresis and in-gel digestion were performed as previously described.^93^ Briefly, cell lysates (10 μL) and secretomes (30 μL) were separated on precast 4–12% gradient gels using the NuPAGE SDS-PAGE system (Thermo Fisher Scientific, USA, #NP0336). Following electrophoresis, gels were fixed in 50% ethanol/3% phosphoric acid solution and stained with Coomassie R250. Subsequently, the gels were washed once in 50 mM ABC and twice in 50 mM ABC/50% ACN, followed by reduction in 10 mM DTT/50 mM ABC for 1 h at 56°C and alkylation in 50 mM iodoacetamide for 45 min at room temperature in the dark. After washing once in 50 mM ABC and twice in 50 mM ABC/50% ACN, gel lanes were cut into 3 bands (for both cell line secretome and cell lysate samples) and each band was cut into ∼1 mm^3^ cubes. Gel cubes were first washed in 50 mM ABC/50% ACN and then dried in vacuum centrifuge for 10 min at 50°C. Following gel cube rehydration by trypsin solution (Promega, 6.25 ng/mL in 50 mM ABC), the gel cubes were covered with 50 mM ABC and incubated overnight at 25°C. Peptides were isolated from the gel cubes with 5% FA/50% ACN (twice) and 1% formic acid (FA) (once). In a vacuum centrifuge at 60°C the extracts were concentrated before LC-MS/MS after which volumes were adjusted to 50 μL with 0.05% FA into LC autosampler vials after filtering through a spin filter of 0.45 μm.^94^

#### LC-MS/MS proteomic analysis

Peptides were separated using an Ultimate 3000 nanoLC-MS/MS system (Thermo Fisher Scientific, USA) equipped with a 40 cm × 75 μm ID fused silica column custom packed with 1.9 μm 120 Å ReproSil Pur C18 aqua (Dr Maisch GMBH, Ammerbuch-Entringen, Germany). After injection, peptides were trapped at 10 μL/min on a 10 mm × 100 μm ID trap column packed with 5 μm 120 Å ReproSil Pur C18 aqua in buffer A (buffer A: 0.1% formic acid in MQ; buffer B: 80% ACN + 0.1% formic acid in MQ) and separated at 300 nL/min in a 10–40% buffer B gradient in 90 min (130 min inject-to-inject) at 35°C. Eluting peptides were ionized at a potential of +2 kVa into a Q Exactive mass spectrometer (Thermo Fisher Scientific, USA). Intact peptide masses were measured at resolution 70.000 (at m/z 200) in the orbitrap using an AGC target value of 3 × 106 charges. The top 10 peptide signals (charge-states 2+ and higher) were submitted to MS/MS In the HCD (higher-energy collision) cell (1.6 m/z isolation width, 25% normalized collision energy) using an AGC target value of 1 × 106 charges an underfill ratio of 0.5% and a maxIT of 60 ms at resolution 17.500 (at m/z 200).

#### Protein identification and database searching

The FASTA protein sequence file of UniProtKB/Swiss-Prot human database (released in January 2021 with 42,383 entries) was used to match theoretical fragmented ions to the measured spectra using the MaxQuant search engine (version 1.6.10.43).^95^ Two missed cleavages were allowed. Peptide modification was set to cysteine carbamidomethylation, and methionine oxidation and N-terminal acetylation were added as variable modifications in the search parameters. The maximally allowed mass error for the precursor mass (MS) was 4.5 ppm and for and fragment mass (MS/MS) was 20 ppm, respectively. Both peptide and protein identifications were filtered at a false discover rate (FDR) 0.01 using the target-decoy strategy (default in MaxQuant). The mass spectrometry proteomics data have been deposited to the ProteomeXchange Consortium via the PRIDE^96^ partner repository with the dataset identifier PXD041042.

#### Statistical analyses and data mining of LC-MS/MS data

Quantification of proteins was done using spectral counting, which is the sum of all MS/MS spectra for every detected protein.^97^ Spectral counts for known proteins in a sample were normalized to the sum of spectral counts for that sample and subsequently multiplied by the mean of the sum for all samples. Normalization and statistical testing were performed using the R package countdata. Differential statistical analyses of samples was performed on the normalized spectral counts per sample using the inverted beta-binomial test^98^ to compare protein expression between the paired control and treatment samples. Statistical significance was assessed with a p value of ≤0.05 and log2FC < −0.1 or >0.1. Statistical enrichment analysis for Gene Ontology (GO) Biological Processes and Cellular Compartment was performed using g:Profiler^90^ (*p*-value_adj_ ≤ 0.05, version e104_e.g.,51_p15_2719230). KEGG pathway enrichment analysis was performed using Enrichr (*p*-value_adj_ ≤ 0.05, KEGG_2021_Human).^91^ Prediction of classically secreted, intracellular, transmembrane and unconventionally secreted proteins was performed using OutCyte.^31^

#### xMAP (multiplex) assays

For validation of LC-MS/MS findings, MDA-MB-231 cells were seeded in a 96-well plate (20,000 cells) in serum-free or serum-containing conditions and were allowed to reach ∼90% confluency over 24 h. Cells were subsequently treated with media, CRT(2.5μΜ) or CRT(1μΜ). MDA-MB-468, MDA-MB-436, MDA-MB-453, BT549, HCC1806, HCC38, HCC70, HCC1143 and HCC1937 cells were seeded in a 96-well plate (approx. 20,000 cells) and were allowed to reach ∼90% confluency over 24 h. Cells were subsequently treated with 100 μL of (a) serum containing DMSO control (b) 2.5 μM CRT0066101 for 2 h followed by replacement with serum containing medium for 22 h. Conditioned medium was collected and stored at −80°C, pending analysis. For validation of PKD inhibition and stimulation conditions, TNBC cells were treated with (a) DMSO control (b) 2.5 μM CRT0066101 for 2 h (c) 1 μM PMA for 15 min (d) 2.5 μM CRT0066101 for 2 h followed by 1 μM PMA for 15 min. PMA was purchased from Merck Millipore (#P1585). Following the addition of Protavio lysis buffer (Protavio, Athens, Greece), cells were lysed by freezing/thawing and cellular debris was removed by centrifugation at 2,700 g for 20 min. Cell lysates were used for protein quantification using a BCA Protein Assay Kit (Pierce, Thermo Scientific, USA, #23225) to ensure equal amounts of protein pre- and post-CRT0066101 treatment.

xMAP assays were performed on a Luminex FLEXMAP 3D platform (Luminex, USA), using custom-developed protein panels for STC-1, LIF, GM-CSF, M-CSF, IL-11, MMP-1, MMP-13. A custom-developed xMAP assay was used to detect pS876 PKD2 phosphorylation. Custom antibody-coupled beads were technically validated as described before.^99^

#### ELISA

For quantification of TNC, analysis was performed using a commercially available human TNC (Abcam, UK, #ab213831) enzyme-linked immunosorbent assay (ELISA) kit, according to the manufacturer’s instructions.

#### Transient siRNA transfection

Cells were transfected with Lipofectamine RNAiMAX (Thermo Fisher Scientific, USA, #13778030) according to manufacturer’s instructions. siRNAs were purchased from GE Healthcare Dharmacon Inc (spNon: #D-001810–10, spPKD3: #L-005029, spPKD2: #L-004197). Cells were transfected with siRNAs for PKD2, PKD3 and both for PKD2 and PKD3. Following 48 h of transfection, cells were incubated for 24 h in serum containing medium and secretome samples were collected. Cell lysates were used for protein quantification using a BCA Protein Assay Kit (Pierce, Thermo Scientific, USA, #23225) to ensure equal amounts of protein pre- and post-knockdown.

#### Spheroid formation

MDA-MB-231 cells were used for the multicellular spheroid formation and invasion assay. In brief, Matrigel (Corning, USA, #356231) was thawed overnight on ice. After 24 h, cells were washed with PBS, harvested with trypsin and resuspended in fresh medium. In 100 μL of cold medium, 1000 cells were seeded in 96-well ultra-low binding plates supplemented with v/v 2.5% Matrigel. All reagents were kept at 4°C and all steps were performed on ice to avoid premature polymerization of Matrigel. After seeding the cells, the plates were centrifuged at 125 G for 10 min at 8°C. The cells were kept at 37°C for 48 h to allow for spheroid formation.

#### 3D invasion assay

Neutralization of the collagen (10 mg/mL, FibriCol, Cell Systems, USA, #5133) was performed on ice by adding ice-cold 125 μL 10X PBS, 125 μL 0.1 M NaOH and 25 μL 0.1 M HCl to 1 mL collagen solution. The reaction was mixed well between each step and then 1x PBS was added until a concentration of 2 mg/mL was reached. Bubbles in the collagen were prevented by centrifugation at 500 G for 3 min at 4°C. The spheroids in the 96 well ultra-low adhesion plate (Thermo Scientific, USA, #174929) were incubated on ice for 10 min. Then 100 μL of neutralized collagen was carefully added to the wells to reach the final collagen concentration of 1 mg/mL. To ensure that the spheroids were centered and to remove excess air bubbles, the plates were centrifuged at 300 g for 3 min at 4°C. The plate was then placed in a 37°C incubator for 60 min to polymerize the collagen. After polymerization, the conditioned medium collected as described in the “transient siRNA transfection” STAR methods section (siRNA control treated (spNon: #D-001810–10) and siPKD2 treated (spPKD2: #L-004197)), was carefully mixed 1:1 with DMEM supplemented with 10% FCS and added to the spheroids. After 2 days, the conditioned medium was renewed. As a control, the spheroids were incubated with DMEM supplemented with 10% FCS. The spheroids were imaged every 3 h for 5 days at 10× magnification after addition of collagen using the IncuCyte S3 and the invasion and core area were analyzed using the IncuCyte S3 software. Normalised invasive and core area were calculated by dividing the respective invasive/core area of siRNA treated spheroids by the mean invasive/core area of medium-treated spheroids.

### Quantification and statistical analysis

Statistical analyses were performed using using R (version 4.0.3) and R Studio (version 2022.12.0) software (R Foundation for Statistical Computing, Vienna, Austria, RRID:SCR_001905). Mean values and standard deviations are shown in figures that were generated from three to four replicates of biological experiments. Student’s *t* test two-tail statistics analysis was used to compare multiplex and ELISA data samples with controls following normality testing. For 3D invasion assay results, spheroid invasive area and core area were compared across conditions using a one-way ANOVA, followed be Tukey’s multiple comparisons. p values ≤0.05 were considered statistically significant and are reported within the Figures as: ∗p < 0.05, ∗∗p < 0.01, ∗∗∗p < 0.001. Groups without statistical significance were marked “ns”.

## Supplemental information

Document S1. Figures S1–S11 and Table S5

Table S1. Identified proteins per secretome sample and biological replicate, related to Figure 1 UniprotID, Gene Name and Normalized count provided for each protein identification per sample.

Table S2. Regulated secretome proteins per comparison, related to Figure 1 UniprotID, Gene Name, Normalized count for each protein identification per sample, Fold change and p-value provided.

Table S3. Overlapping secretome proteins per comparison, related to Figure 1 UniprotID, Gene Name, Normalized count for each protein identification per sample, Fold change and p-value provided.

Table S4. Identified proteins per cell lysate sample and biological replicate, related to Figure 1 UniprotID, Gene Name and Normalized count provided for each protein identification per sample.
