# Supplementary material for: Protein kinase D drives the secretion of invasion mediators in triple-negative breast cancer cell lines
Source: iScience. 2024 Jan 17;27(2):108958. doi: 10.1016/j.isci.2024.108958 (PMC10844833; doi:10.1016/j.isci.2024.108958)
Supplement: Document S1. Figures S1–S11 and Table S5 [file mmc1.pdf]

iScience, Volume *27*

Supplemental information

Protein kinase D drives the secretion of invasion mediators in triple-negative breast cancer cell lines

Alexia Gali, Irene V. Bijnsdorp, Sander R. Piersma, Thang V. Pham, Elena Gutiérrez-Galindo, Fiona Kühnel, Nikos Tsolakos, Connie R. Jimenez, Angelika Hausser, and Leonidas G. Alexopoulos

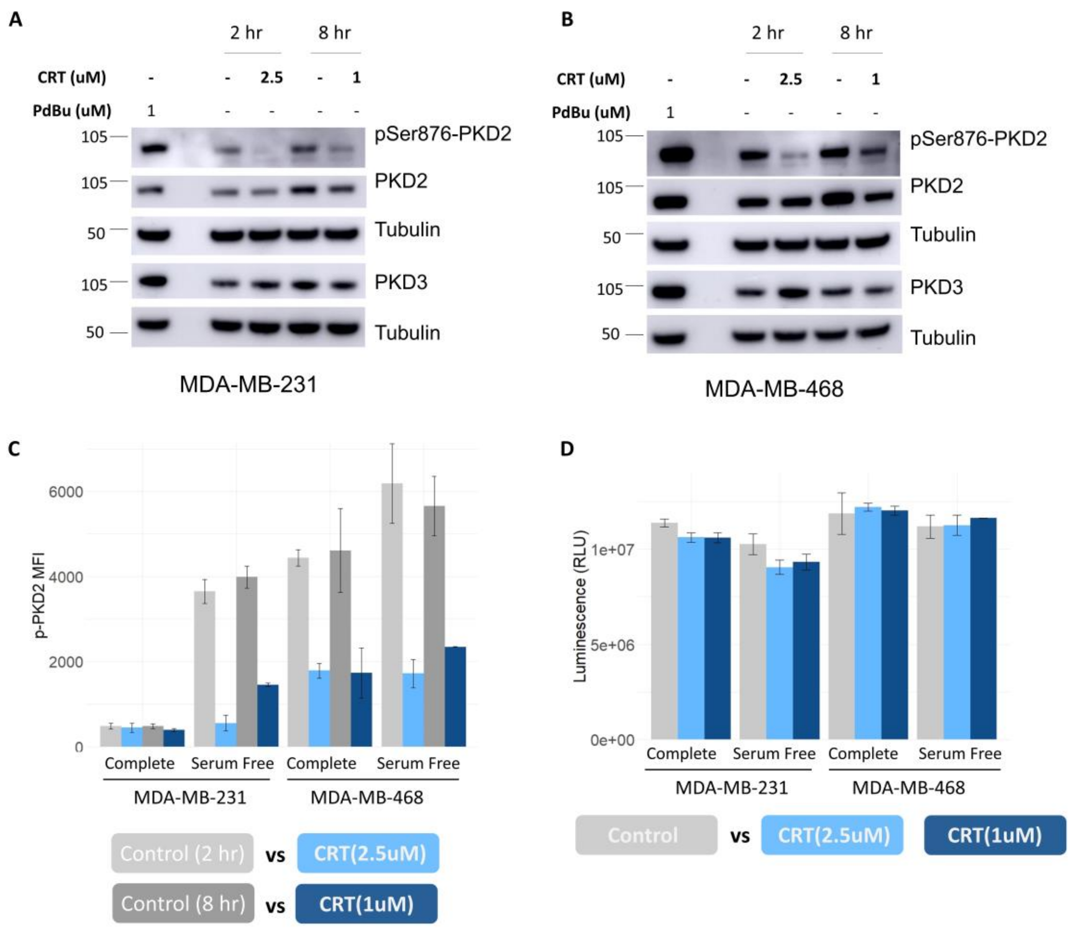

Figure S1 (Related to Figure 1). Optimization of conditions used for LC-MS/MS sample analysis.

A and B, Western blot of (**A**) MDA-MB-231 and (**B**) MDA-MB-468 cells, comparing CRT(2.5uM) and CRT(1uM) with the respective control samples under serum free conditions. PdBu treatment was used as positive control of PKD autophosphorylation. Immunoblotting was conducted, and membranes were probed with specific antibodies as indicated. Tubulin was used as loading control. **C,** xMAP assay for the detection of pS876 PKD2 in MDA-MB-231 and MDA-MB-468 cells, comparing CRT(2.5uM) and CRT(1uM) with the respective control samples, under serum free and serum containing conditions. Data are reported as mean Median Fluoresence Intensity (MFI) values obtained from three biological replicates and error bars show standard deviation. **D,** Cell viability assessed using the CellTiter-Glo reagent comparing CRT(2.5uM) and CRT(1uM) with control under serum free and serum containing conditions. Data are reported as mean luminescence values (RLU) obtained from three technical replicates and error bars show standard deviation.

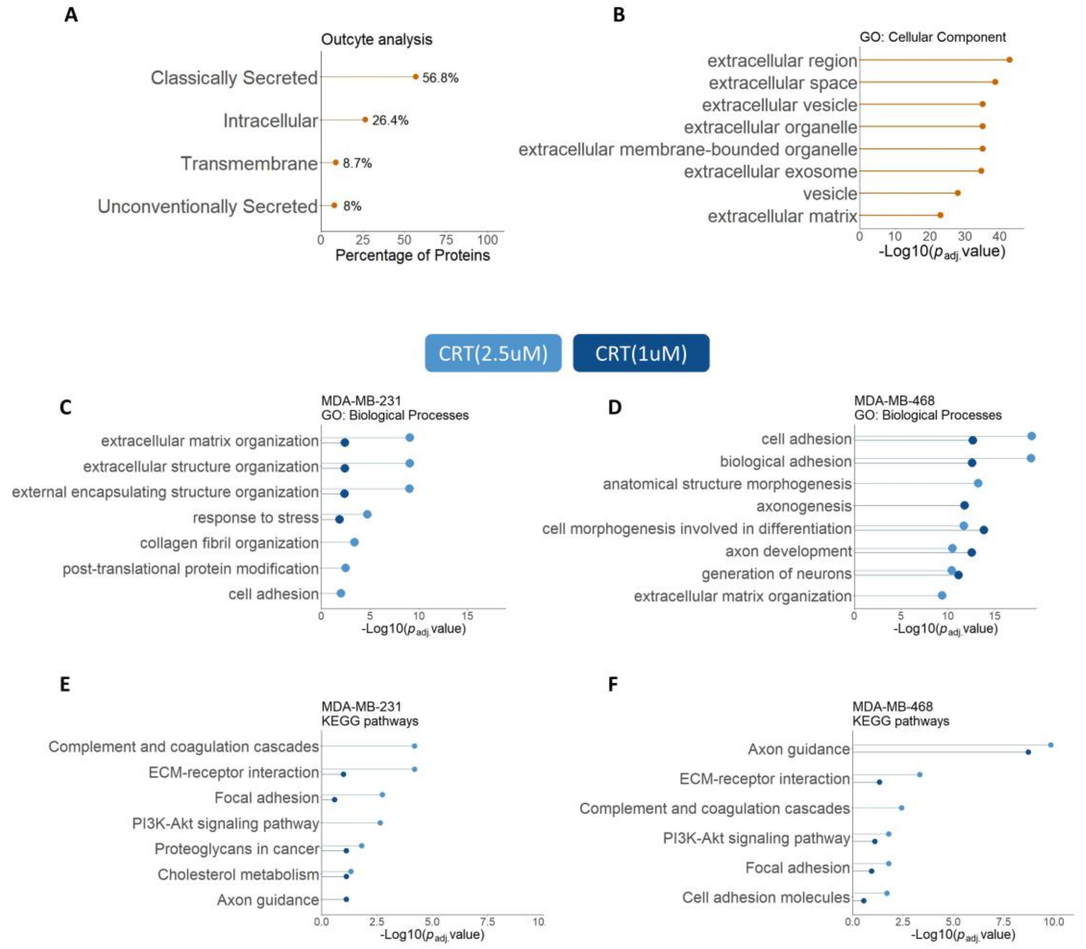

Figure S2 (Related to Figure 2). PKD inhibition reduces the secretion of cell adhesion and extracellular matrix proteins in TNBC.

A, Percentage of secreted, intracellular, transmembrane and unconventionally secreted proteins predicted by the Outcyte algorithm in the downregulated proteins of CRT(2.5μM) and CRT(1μM) in both MDA-MB-231 and MDA-MB-468 cells. **B,** Top Gene Ontology (GO) cellular compartment terms identified from the downregulated proteins of CRT(2.5μM) and CRT(1μM) in both MDA-MB-231 and MDA-MB-468 cells. **C** and **D,** Top GO biological processes enriched in the downregulated proteins identified in **(C)** MDA-MB-231 and **(D)** MDA-MB-468 cells following CRT(2.5μM) and CRT(1μM). **E** and **F,** Top KEGG pathways enriched in the downregulated proteins identified in **(E)** MDA-MB-231 and **(F)** MDA-MB-468 cells following CRT(2.5μM) and CRT(1μM). X-axis indicates the enrichment scores [-log10 (adjusted p value), p-value cut-off of 0.05] for each term and y-axis the enriched term.

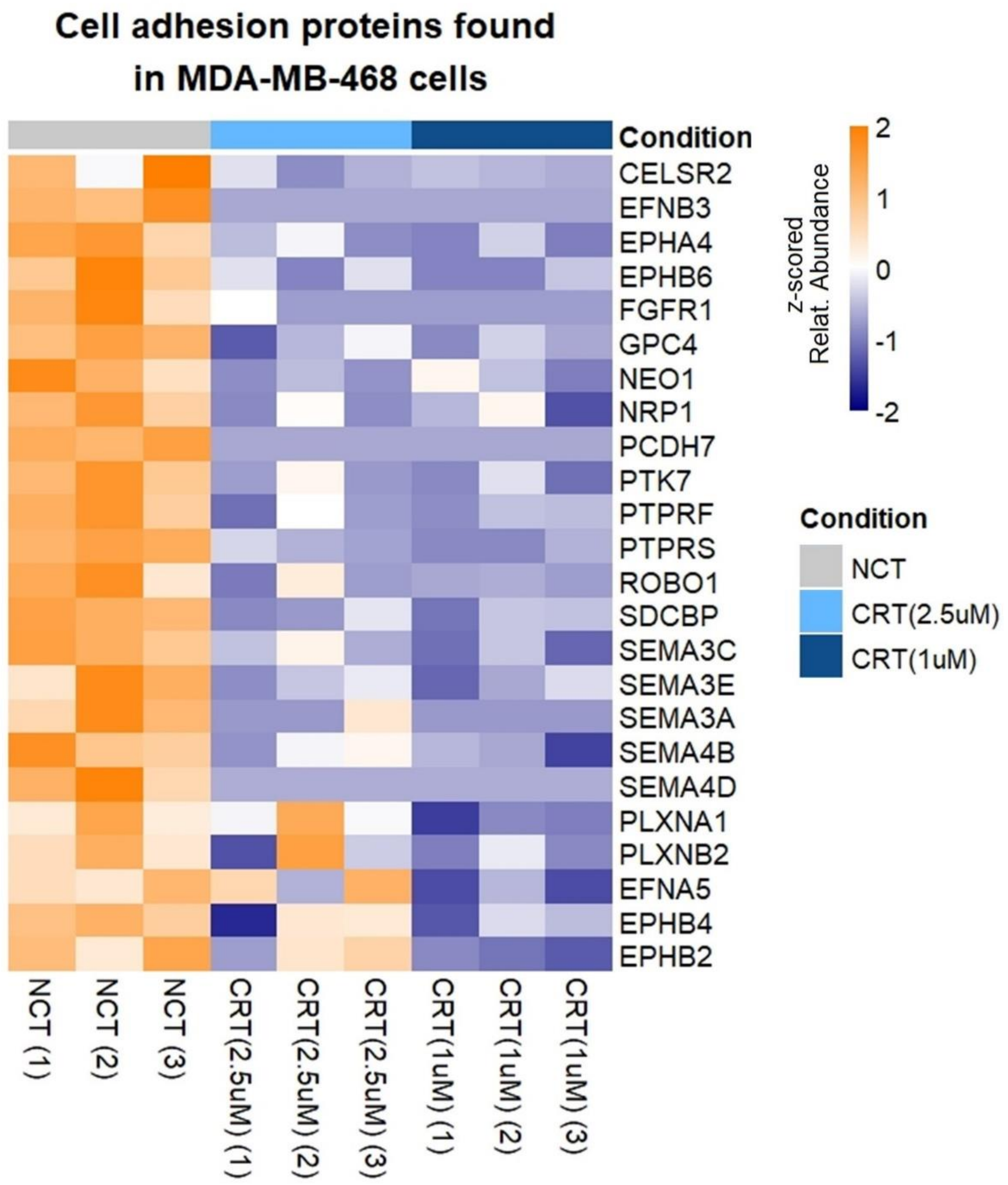

Figure S3 (Related to Figure 2). PKD inhibition reduces the secretion of cell adhesion proteins in the MDA-MB-468 cell line.

Heatmap of Z-scores for protein abundances of selected cell adhesion-annotated proteins found in MDA-MB-468 cells following CRT(2.5uM) and CRT(1uM). Each column represents a biological replicate (n = 3) of control or CRT(2.5uM) or CRT(1uM). Relative protein abundances are shown from low (blue) to high (orange).

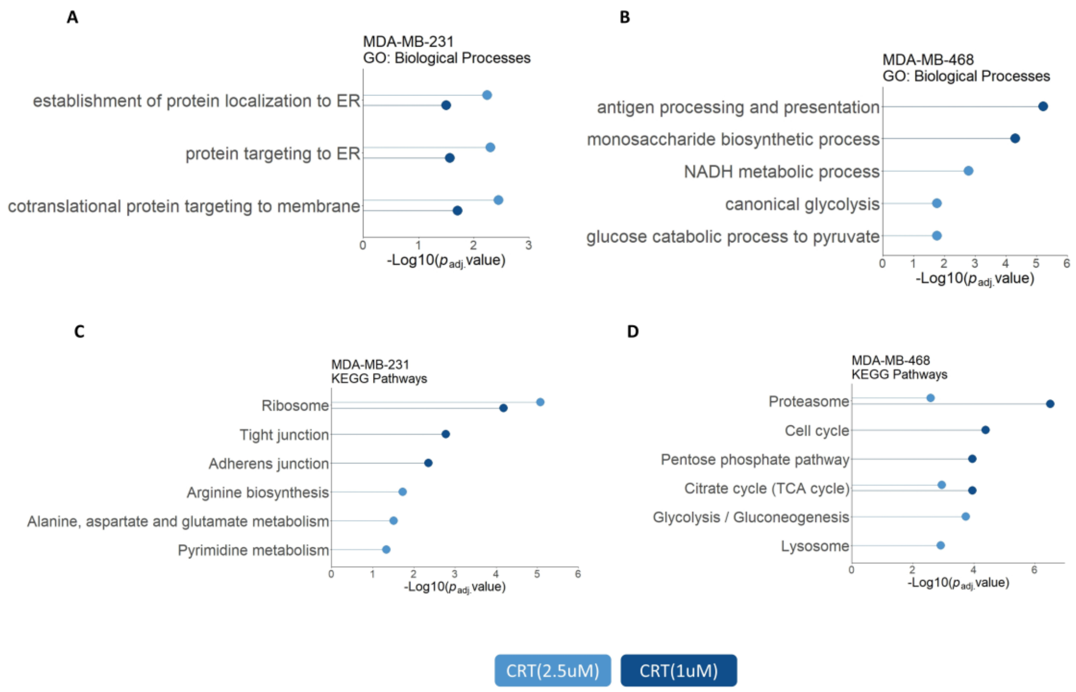

Figure S4 (Related to Figure 1). PKD inhibition increases the secretion of proteins related to metabolism in TNBC

A and B, Top GO biological processes enriched in the upregulated proteins identified in (**A**) MDA-MB-231 and (**B**) MDA-MB-468 cells following CRT(2.5μM) and CRT(1μM). **C and D,** Top KEGG pathways enriched in the upregulated proteins identified in **(C)** MDA-MB-231 and **(D)** MDA-MB-468 cells following CRT(2.5μM) and CRT(1μM). X-axis indicates the enrichment scores [-log10 (adjusted p value), p-value cut-off of 0.05] for each term and y-axis the enriched term

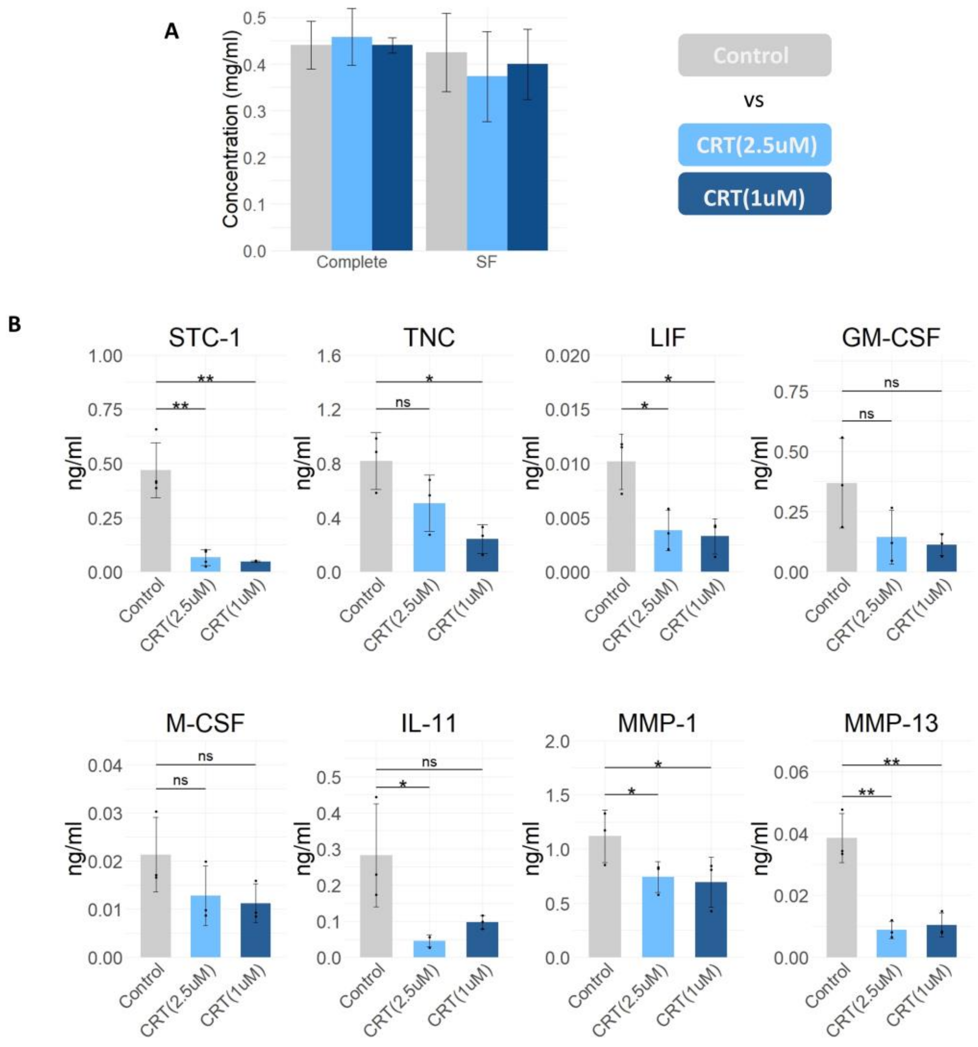

Figure S5 (Related to Figure 2). PKD inhibition reduces the secretion of TNBC invasion mediators under serum free conditions.

A, Concentration of cellular lysates of MDA-MB-231 cells, under complete media and serum free (SF) conditions, after treatment with control, CRT(2.5uM) or CRT(1uM). **B,** Quantification of eight selected secretome proteins in the MDA-MB-231 cells under serum free conditions. Protein secretion was quantified in secretome samples using multiplex assays for STC-1, LIF, GM-CSF, M-CSF, IL-11, MMP-1, MMP-13 and ELISA for TNC. Data are reported as mean of three or four biological replicates and error bars show standard deviation. *P* values were assessed by unpaired, two-tailed Student's t-test. *∗p < 0.05; ∗∗p < 0.01; ∗∗∗p < 0.001; ns: not significant.*

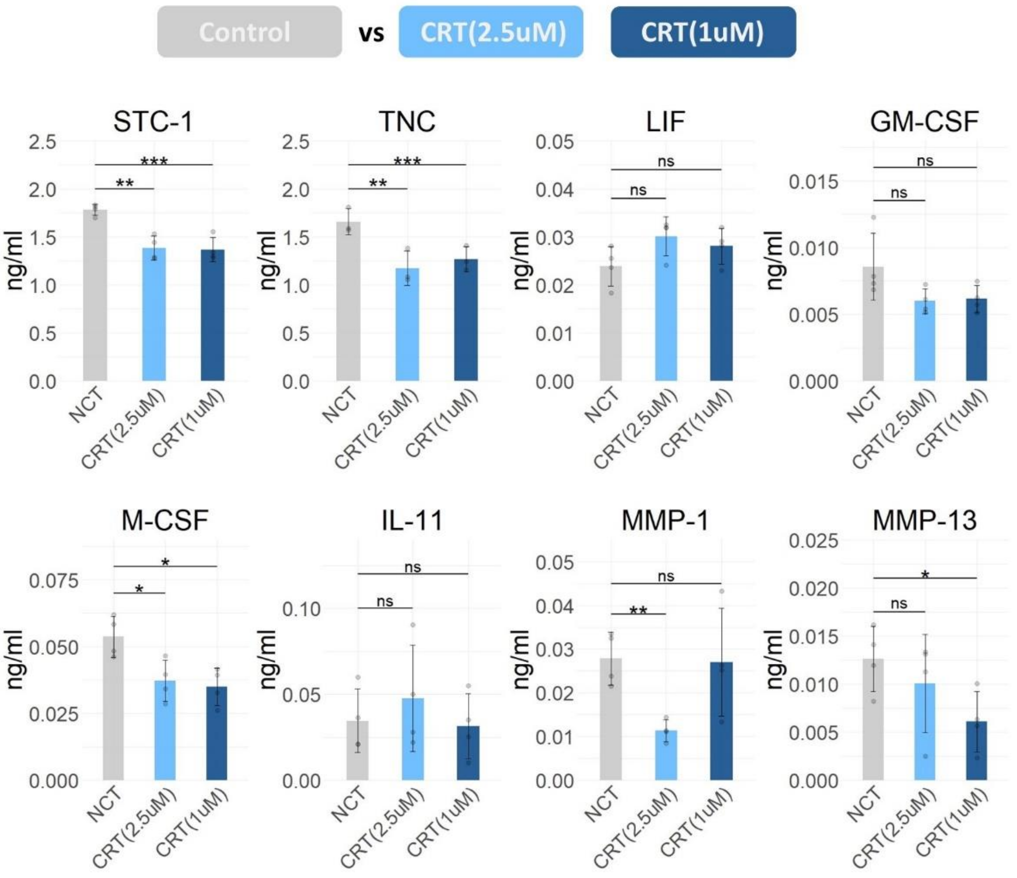

Figure S6 (Related to Figure 2). PKD promotes secretion of invasion mediators by promoting protein synthesis and/or differential secretion.

Quantification of eight selected secretome proteins in the cell lysates of MDA-MB-231 cells. Levels of proteins were quantified in cell lysate samples using multiplex assays for STC-1, LIF, GM-CSF, M-CSF, IL-11, MMP-1, MMP-13 and ELISA for TNC. Data are reported as mean of three or four biological replicates and error bars show standard deviation. *P* values were assessed by unpaired, two-tailed Student's t-test. *$*p < 0.05$; $**p < 0.01$; $***p < 0.001$; ns: not significant.*

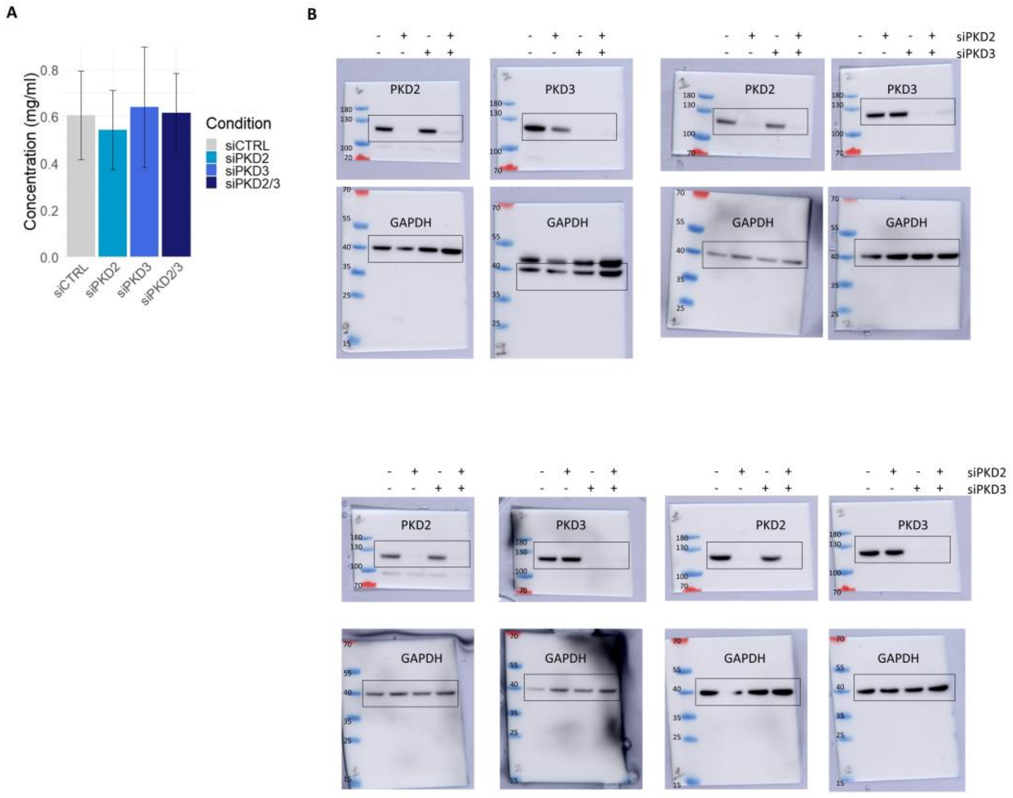

Figure S7 (Related to Figure 3). Confirmation of PKD2, PKD3 and double PKD2/PKD3 knockdown.

A, Concentration of cellular lysates of MDA-MB-231 cells following transfection with non-targeting control siRNA (siCTRL), PKD2 siRNA (siPKD2), PKD3 siRNA (siPKD3), and both PKD2 and PKD3 siRNA (siPKD2/3). Data are reported as mean of four biological replicates and error bars show standard deviation. **B,** Uncropped Blots from Figure 3. Immunoblotting was conducted, and membranes were probed with specific antibodies as indicated. GAPDH was used as loading control.

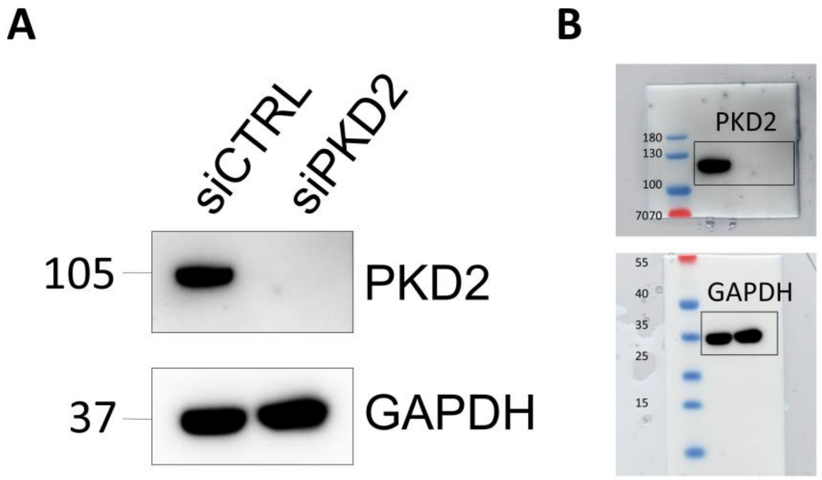

Figure S8 (Related to Figure 4). Confirmation of PKD knockdown.

A, Western blot comparing siCTRL and siPKD2 in MDA-MB-231 cells in preparation for the collection of conditioned media to be used in 3D invasion assay. Immunoblotting was conducted, and membranes were probed with specific antibodies as indicated. GAPDH was used as loading control. **B,** Uncropped blots of the presented Western blot.

Cell Line	Subtype	Primary/Metastasis	PRKD2 TPM	PRKD3 TPM
HCC1143	BasalA	Primary	5.39	3.65
HCC1806	BasalA	Primary	5.60	4.35
HCC1937	BasalA	Primary	5.52	3.20
HCC70	BasalA	Primary	5.28	4.46
MDA-MB-468	BasalA	Metastasis	5.38	5.08
BT549	BasalB	Primary	3.90	4.47
HCC38	BasalB	Primary	5.06	6.09
MDA-MB-231	BasalB	Metastasis	3.88	4.33
MDA-MB-436	BasalB	Metastasis	3.99	4.69
MDA-MB-453	Luminal	Metastasis	4.90	0.41

Table S5 (Related to Figure 5). Panel of TNBC cell lines.

TNBC cell line characteristics, including TNBC subtype and original site of cell line establishment (Primary/Metastasis). Transcript per million (TPM) values of PKD2 and PKD3 expression obtained from DepMap (Public 22Q4).

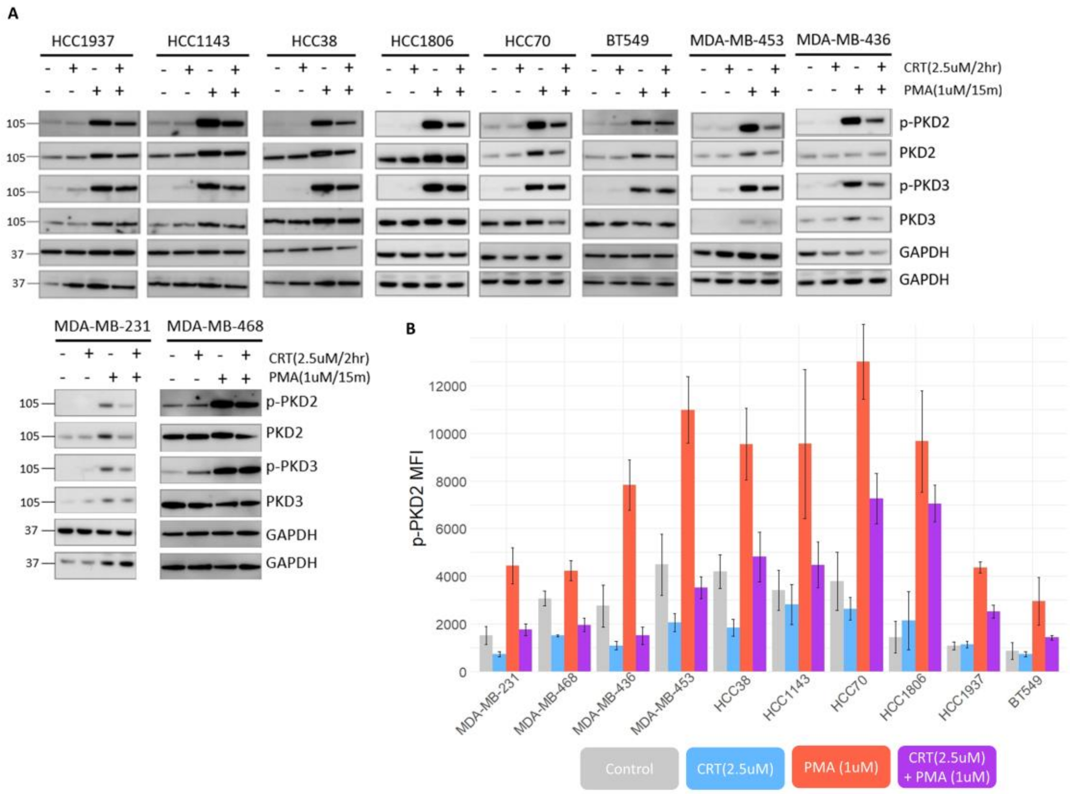

Figure S9 (Related to Figure 5). Confirmation of PKD inhibition and stimulation conditions in a panel of 10 TNBC cell lines.

A, Western blot comparing CRT(2.5uM), PMA(1uM) and CRT(2.5uM) followed PMA(1uM) in TNBC cell line panel. Immunoblotting was conducted, and membranes were probed with specific antibodies as indicated. GAPDH was used as loading control. Uncropped blots found in Supplementary Figure S8. **B,** xMAP assay for the detection of pS876 PKD2 in TNBC cell line panel using the same conditions as in Western Blot. Data are reported as mean Median Fluoresence Intensity (MFI) values obtained from three to four biological replicates and error bars show standard deviation.

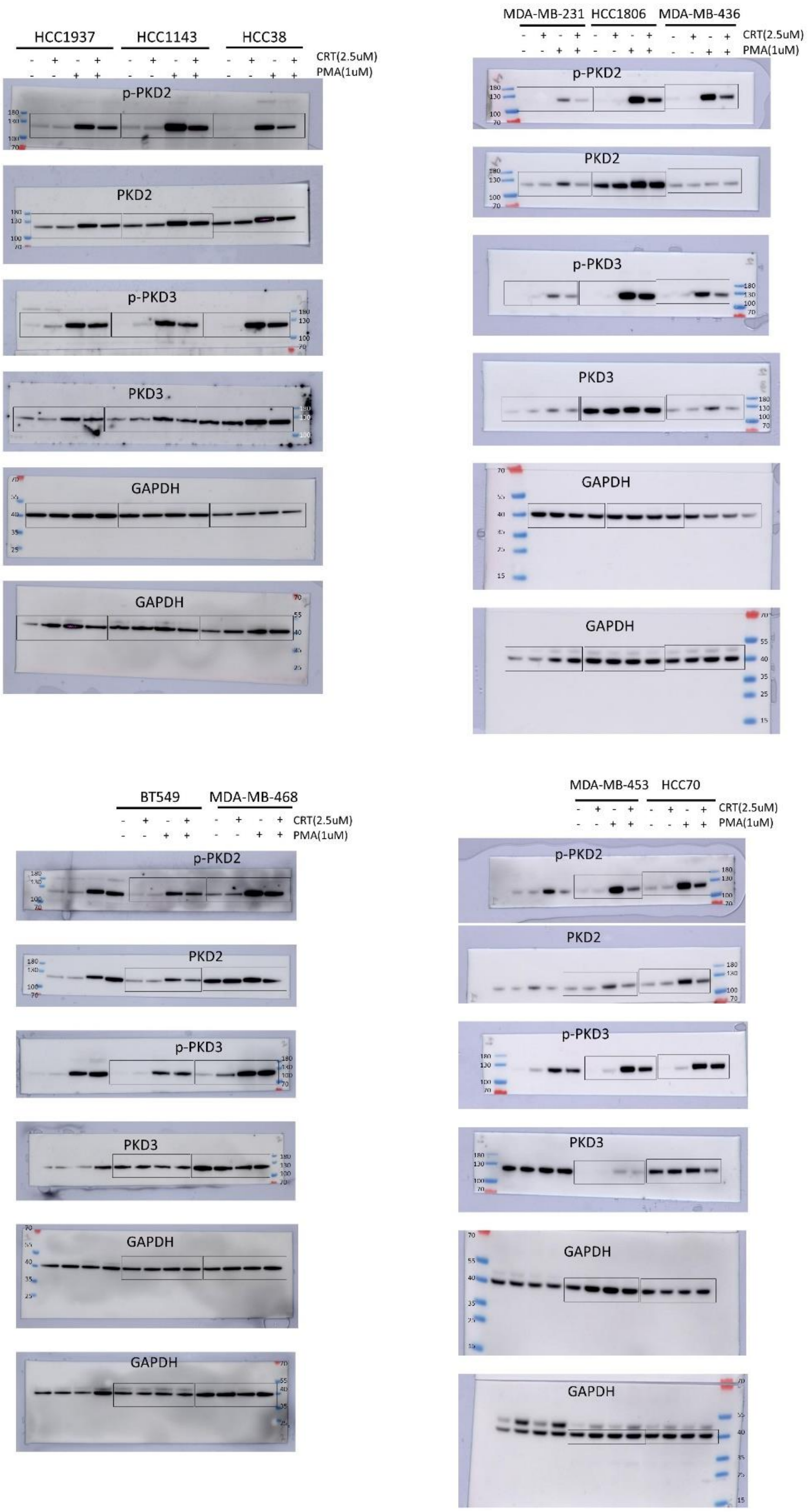

Figure S10 (Related to Figure 5 and Figure S9). Uncropped Blots from Figure S9. Immunoblotting was conducted, and membranes were probed with specific antibodies as indicated. GAPDH was used as loading control**.**

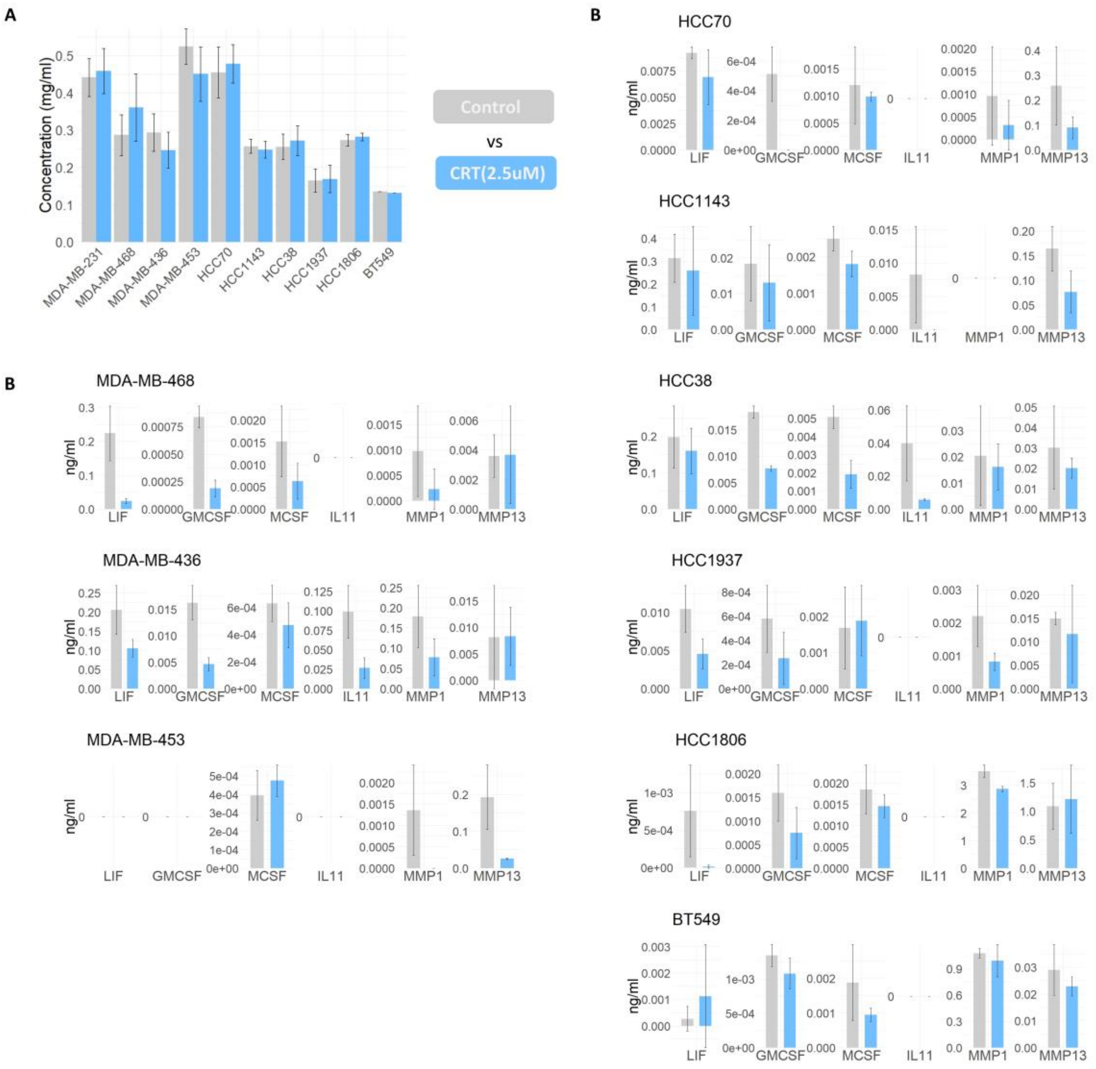

Figure S11: **(Related to Figure 5). PKD regulates the secretion of invasion mediators in a panel of TNBC cell lines.**

A, Concentration of cellular lysates of the ten cell lines, after treatment with DMSO control or CRT(2.5uM). Data are reported as mean of four biological replicates and error bars show standard deviation. **B,** Protein measurements in the secretome of TNBC cell lines following CRT(2.5uM), compared to DMSO control. Levels of proteins were quantified in secretome samples using multiplex assays for LIF, GM-CSF, M-CSF, IL-11, MMP-1 and MMP-13. Data are reported as mean of three or four biological replicates and error bars show standard deviation. Protein measurements in the secretome of TNBC cell lines that showed statistically significant reduction following CRT(2.5μM) can be found in Figure 4B. Protein measurements for MDA-MB-231 cells can be found in Figure 2D.
